# CD142 Identifies Neoplastic Desmoid Tumor Cells, Uncovering Interactions Between Neoplastic and Stromal Cells That Drive Proliferation

**DOI:** 10.1158/2767-9764.CRC-22-0403

**Published:** 2023-04-25

**Authors:** Mushriq Al-Jazrawe, Steven Xu, Raymond Poon, Qingxia Wei, Joanna Przybyl, Sushama Varma, Matt van de Rijn, Benjamin A. Alman

**Affiliations:** 1 Hospital for Sick Children, Program in Developmental & Stem Cell Biology, Toronto, Ontario, Canada.; 2 Laboratory Medicine & Pathobiology, University of Toronto, Toronto, Ontario, Canada.; 3 Department of Pathology, Stanford University School of Medicine, Stanford, California.; 4 Department of Orthopedic Surgery, Duke University, Durham, North Carolina.; 5 Regeneration Next Initiative, Duke University, Durham, North Carolina.

## Abstract

The interaction between neoplastic and stromal cells within a tumor mass plays an important role in cancer biology. However, it is challenging to distinguish between tumor and stromal cells in mesenchymal tumors because lineage-specific cell surface markers typically used in other cancers do not distinguish between the different cell subpopulations. Desmoid tumors consist of mesenchymal fibroblast-like cells driven by mutations stabilizing beta-catenin. Here we aimed to identify surface markers that can distinguish mutant cells from stromal cells to study tumor–stroma interactions. We analyzed colonies derived from single cells from human desmoid tumors using a high-throughput surface antigen screen, to characterize the mutant and nonmutant cells. We found that CD142 is highly expressed by the mutant cell populations and correlates with beta-catenin activity. CD142-based cell sorting isolated the mutant population from heterogeneous samples, including one where no mutation was previously detected by traditional Sanger sequencing. We then studied the secretome of mutant and nonmutant fibroblastic cells. PTX3 is one stroma-derived secreted factor that increases mutant cell proliferation via STAT6 activation. These data demonstrate a sensitive method to quantify and distinguish neoplastic from stromal cells in mesenchymal tumors. It identifies proteins secreted by nonmutant cells that regulate mutant cell proliferation that could be therapeutically.

**Significance:**

Distinguishing between neoplastic (tumor) and non-neoplastic (stromal) cells within mesenchymal tumors is particularly challenging, because lineage-specific cell surface markers typically used in other cancers do not differentiate between the different cell subpopulations. Here, we developed a strategy combining clonal expansion with surface proteome profiling to identify markers for quantifying and isolating mutant and nonmutant cell subpopulations in desmoid tumors, and to study their interactions via soluble factors.

## Introduction

Stromal cells of the tumor microenvironment play an important role in disease progression and therapeutic response. However, the investigation of their role in mesenchymal tumors is hampered by difficulties distinguishing them from the neoplastic cells. Desmoid tumors consist of proliferative and infiltrative mesenchymal fibroblast-like cells within a collagenous extracellular matrix. Their clinical behavior is unpredictable, ranging from locally aggressive disease to spontaneously regressing lesions (1, 2). There are no universally effective therapies (3). Somatic mutations activating beta-catenin, the effector molecule of canonical Wnt signaling, is a cardinal finding in desmoid tumors (4). It is estimated that over 90% of desmoid tumors exhibit mutations in the gene encoding beta-catenin (*CTNNB1*) or in adenomatous polyposis coli (*APC*), which phosphorylates beta-catenin and tags it for subsequent degradation (5, 6). The somatic mutations observed in the *CTNNB1* gene alter any one of four serine or threonine residues found in its third exon. These residues are required for efficient beta-catenin degradation. As a result of the mutated residue, beta-catenin protein accumulates in the cell, where it can translocate into the nucleus, altering gene expression (7). The two most common somatic *CTNNB1* mutations detected are T41A and S45F, accounting for approximately 50% and 30% of all reported patients with sporadic desmoid tumor, respectively (6, 8–11). Some desmoid tumors were thought to have no detectable mutations and are labeled wildtype when mutational analysis was undertaken using Sanger sequencing. Next-generation sequencing demonstrated that the vast majority of these tumors do in fact contain a subpopulation of mutant cells but at too low a frequency to be detected by Sanger sequencing (5, 6). Despite the use of cryomolds to dissect contaminating tissues in these next-generation sequencing efforts, *CTNNB1* mutation was only detectable in a minority of the reads per sample (5). We previously found that desmoid tumors can be derived from Ng2-expressing perivascular cells in mice (12). In that investigation, we also found a population of cells in the murine tumors which did not derive from these tumor-initiating cells. Taken together, this suggests that desmoid tumors exhibit strong intratumoral heterogeneity. Some cells may be non-neoplastic stromal cells infiltrating the tumor mass, which makes Sanger sequencing not suitable to detect *CTNNB1* mutations in a subset of cases.

Tumor-associated fibroblasts predominate in the cellular tumor microenvironment and their role in disease progression is well documented (13, 14). These mesenchymal cells are implicated in regulating the neoplastic phenotype of several tumors including breast cancer (15, 16), squamous cell carcinoma (17), non–small cell lung cancer (18), and scirrhous gastric carcinoma (19) among many others. Tumor-associated fibroblasts can stimulate tumor progression and invasiveness (14, 17, 20), but they can also restrain tumor progression in other contexts (21–23). Recently, they have also been implicated in therapeutic response and drug resistance (24, 25). Little is known about the tumor stroma and its effect on neoplastic cells in mesenchymal tumors. This is in part because investigating the role of the tumor stroma in mesenchymal lesions has been challenging because the neoplastic cells exhibit the same mesenchymal phenotype as the stromal fibroblasts. In fibroproliferative diseases like desmoid tumors, the distinction is doubly challenging as both groups exhibit a fibroblastic phenotype. Surface proteins that differ in their expression between the two groups offer a potential identifying feature. While cell surface profiling of fibroblasts has been conducted previously (26), how the neoplastic and non-neoplastic mesenchymal cells within desmoid tumors compare is not known. We therefore sought to study the expression of surface proteins in single cell–derived samples of desmoid tumors.

Neoplastic and stromal cells can communicate via paracrine signaling (27). This cross-talk can modulate tumor initiation, progression, and invasion (13, 14). Previous studies of tumor–stroma interactions have identified a number of important pathways in tumor development, including TGFβ, NFκB, PDGF, Notch, and Src (14, 20). Variability in behavior of other tumors caused by beta-catenin activation, such as colorectal cancers, is linked to the tumor microenvironment playing a major role in modulating beta-catenin activity and tumor progression (28). While it is well known that cytokines such as TGFβ stimulate fibroblast proliferation, mediated by beta-catenin (29), the role of cell-cell communication via paracrine signaling in beta-catenin–driven desmoid tumors has not been investigated. In this study, we isolated neoplastic and stromal cells using clonal expansion. A high-throughput surface antigen screen was used to analyze the expression of proteins on the cell surface. We used these cells to identify and study cell–cell interaction by paracrine signaling and identified a possible therapeutic target based on disrupting this interaction.

## Materials and Methods

### Human Desmoid Tumor Cell Cultures and Generation of Monoclonal Cell Populations

Human desmoid tumor tissue samples were obtained at time of surgical excision and processed for primary cell culture generation as described previously (30, 31) followed by long-term storage in liquid nitrogen. The use of human material was approved by the Institutional Review Board of the Hospital for Sick Children. Because each primary culture exhibited a different colony-forming capacity, we initially performed limiting dilutions (within a range of 1 to 20 cells per 1 well of a 24-well plate) to determine the best concentration to establish single cell–derived colonies for each line. Each well was visually inspected for the presence of a single colony. A cluster of 32 or more cells at 8 days after seeding was counted as a colony. Cells were maintained at 37°C in 5% CO_2_ in normal culture media consisting of DMEM (Wisent, 319-005-CL, containing 4.5 g/L glucose, with l-glutamine, sodium pyruvate, and phenol red), supplemented with 10% FBS (Wisent, 080-450) and antibiotic/antimycotic solution (Wisent, 450-115-EL). For monoclonal cultures, the media was changed daily. For primary cultures, media was changed three times a week, and the cells were passaged at a 1:3 ratio when confluent. All primary cultures for single cell–derived colony formation were used at or before the third passage. For all other experiments, cells were utilized at under six passages. Cells not immediately used for downstream experiments were stored in liquid nitrogen in freezing media (normal culture media with 10% DMSO).

### Sequencing Analysis

Whole-genomic DNA was extracted from approximately 1 × 10^5^ primary and approximately 1 × 10^4^ clonal expansion cells using DNeasy Blood & Tissue Kit (Qiagen, 69506) as per the manufacturer's instructions with some modifications. Briefly, the cells were pelleted by centrifugation at 800 × *g* for 3 minutes and resuspended in 200 μL PBS. To elute the DNA, 75 and 40 μL of water was added to the spin column containing primary and monoclonal samples, respectively, and left to incubate at room temperature for 4 minutes before elution. PCR amplification of exon 3 of *CTNNB1* was conducted using 100 ng of extracted genomic DNA and the Q5 High-Fidelity Master Mix (New England Biolabs, M0492L) in a 50 μL reaction containing primers designed to amplify the human *CTNNB1* exon 3 region (Forward Primer: TCCAATCTACTAATGCTAATACTGTTTCGTA, Reverse Primer: CATTCTGACTTTCAGTAAGGCAATG). PCR amplification was carried using 2720 Thermal Cycler (Applied Biosystems) with the following program: 98°C for 30 seconds initial denaturation. A total of 30 cycles of 98°C for 10 seconds, 60°C for 30 seconds, and 72°C for 20 seconds. Final extension was carried at 72°C for 2 minutes and the product was held at 4°C. The PCR product was purified by running it through 1.7% agarose gel and using the QIAquick Gel Extraction Kit (Qiagen, 28706) as per the manufacturer's instructions with some modifications. Briefly, the column was incubated with Buffer PE for 5 minutes at room temperature before centrifugation. To elute, the DNA was incubated with 30 μL water for 4 minutes at room temperature. Approximately 150 ng of the PCR product was analyzed by Sanger sequencing (TCAG, Hospital for Sick Children). Initial inspection of the chromatograms using FinchTV (https://digitalworldbiology.com/FinchTV) stratified samples based on the altered nucleotide. All T41A samples displayed an A>G mutation at codon 41, and all S45F samples displayed a C>T mutation at codon 45. Codons 32, 33, and 37 were also inspected but no sample harbored a mutation at these codons. To estimate the mutation frequency, the peak height data of the sequencing chromatograms were then analyzed using QSVanalyzer (32) as per the author's instructions (http://dna.leeds.ac.uk/qsv/). Fibroblasts derived from normal, *CTNNB1*-wildtype, skin tissue were used as a reference sample. For each primary sample analyzed, the PCR and Sanger sequencing analysis were repeated at least twice, and the average value was reported to reduce the effect of PCR stochasticity. When possible, multiple original cryopreserved vials were recovered for the same primary sample to reduce the effect of tumor sampling variability.

### High-throughput Surface Antigen Screen

Cells were analyzed by a flow cytometry–based cell surface antigen screen as described previously (26, 33). Cells were resuspended in 10 mL of PBS, with 2 mmol/L ethylenediaminetetraacetic acid (EDTA) and 2% FBS, and were passed through a 70 μm cell strainer to reduce cell clumping. Colonies of the same genotype were pooled into one cell suspension. Cells from mutant and nonmutant colonies were aliquoted over multiwell plates each containing a unique fluorochrome-tagged antibody targeting one known cell-surface antigen, for a total of 368 antibodies. Gating strategy was conducted as described previously (26, 33). A representative gating example is shown in Supplementary Fig. S12A–S12D. The experiment was repeated twice, once for each mutant subgroup (T41A and S45F). A “hit” in each experiment was considered an antigen with a detectable expression in greater than 50% of the cells in one population and less than 10% in the other. Alternatively, an antigen expressed by more than 50% of cells in one population and differed by more than 10-fold in median fluorescent intensity was also considered as a hit. Surface proteins that were detected as hits in both experimental repeats were considered candidate markers for further study.

### Surface Marker–based FACS

Cells were resuspended in sterile cold staining buffer (0.1% gelatin, 2 mmol/L EDTA in PBS) at a concentration of 1 × 10^7^ cells/mL. PE Mouse anti-human CD142 Clone HTF-1 (BD, 550312), PE Mouse Anti-Human OX40 Ligand (CD252) Clone ik-1 (BD Biosciences, catalog no. 558164, RRID:AB_647195), or Alexa Fluor 647 anti-human Podoplanin Antibody Clone NC-08 (BioLegend, catalog no. 337008, RRID:AB_2162063) were then applied to cell suspension at a 1:20 dilution factor and the antibody-cell mixture was incubated in the dark at 4°C for 45 minutes. The mixture was diluted 5-fold with cold staining buffer and centrifuged at 800 × *g* for 3 minutes. The cell pellet was then resuspended at 1 × 10^7^ cells/mL with staining buffer and kept on ice until cell sorting. Propidium iodide (Thermo Fisher Scientific, P3566) was used to detect dead cells. Representative gating strategy is shown in Supplementary Fig. S12E.

### Conditioned Media Generation and Secretome Profiling

A total of 1.5 × 10^5^ cells were plated in each well of a 6-well plate in normal media. After 24 hours, the media was changed to DMEM containing 1% FBS and the cells were incubated for a further 48 hours. The media was subsequently collected and centrifuged at 800 × *g* for 3 minutes at 4°C. The supernatant was transferred to new tubes and stored at −80°C. Secreted protein detection of 105 different cytokines was carried out using the Human XL Cytokine Array Kit (R&D, ARY022B) as per the manufacturer's instructions. Imaging was conducted using the ChemiDoc MP Imaging System (Bio-Rad) and spot pixel density measurements were performed using ImageJ (RRID:SCR_003070) via the Protein Array Analyzer module. For phosphorylated kinase detection after conditioned media treatment, we carried out phosphokinase detection of 43 proteins with Proteome Profiler Human Phospho-Kinase Array (R&D, ARY003B) per the manufacturer's instructions.

### Proliferation Assays

For coculture studies, 1.5 × 10^5^ cells were seeded onto sterile glass cover slips in the bottom chamber of a well in a 6-well plate divided by an 8.0 μm pore size Transwell permeable support (Corning, 3428). Twenty-four hours before staining, the BrdU reagent (Invitrogen) was added to the culture media at a 1:100 dilution. The following day, the cells were fixed with ice-cold methanol for 15 minutes at −20°C. The cells were then washed with PBS for 3 minutes, repeated for a total of three times. Permeablization was carried out using the FixDenat solution (Roche, 11647229001) for 30 minutes at room temperature. The cells were then washed with PBS three times and blocked using a blocking solution of 2% horse serum, 2% donkey serum, and 2% BSA in PBS-T (PBS with 0.1% Tween-20). Following PBS-T washing, Anti-BrdU (Roche, catalog no. 11170376001, RRID:AB_514483) antibody was added at a 1:20 dilution and the samples were incubated for 1 hour at 37°C. Biotinylated horse anti-mouse IgG secondary antibody (Vector Laboratories, catalog no. BA-2000, RRID:AB_2313581) was used at 1:200 dilution for 45 minutes at room temperature. The VECTASTAIN Elite ABC-HRP Kit (Vector Laboratories, PK-6100) was used to amplify the signal followed by DAB Peroxidase [horseradish peroxidase (HRP)] Substrate Kit (with Nickel), 3,3′-diaminobenzidine (Vector Laboratories, SK-4100) as per the manufacturer's instructions. The samples were stained briefly with Mayer's Hematoxylin for 1 minute and washed with running tap water for 1 minute. The cover slips were mounted onto glass slides using VECTASHIELD Antifade Mounting Medium (Vector Laboratories, H-1000). The number of positive nuclei to total nuclei were counted in 10 10X fields. For all other proliferation studies reported here, the colorimetric BrdU incorporation assay (Roche, 11647229001) was used as per the manufacturer's instructions.

### Real-time Quantitative PCR

RNA was extracted from approximately 1.5 × 10^5^ cells using TRIzol Reagent (Invitrogen, 15596-018) as per the manufacturer's instructions. Approximately 1 μg of RNA was used for first-strand synthesis using SuperScript III First-Strand Synthesis System (18080-051) with Oligo(dT)_20_ primers (Invitrogen, 18418-020). Real-time PCR reactions were conducted in the StepOnePlus system (Applied Biosystems) using SYBR Select Master Mix (Applied Biosystems, 4472919) as per the manufacturer's instructions in 25 μL reaction volume. Supplementary Table S5 lists the primers used in these experiments. Relative gene expression levels were calculated using the ΔΔ*C*_t_ method.

### Immunoblotting

Protein lysates were generated using Reporter Lysis Buffer (Promega, E397A). Approximately 10 μg of protein was boiled for 10 minutes in Laemmli buffer with 5% β-mercaptoethanol and 2% SDS. The reduced proteins were resolved on a 7.8% polyacrylamide gel. After transfer to a nitrocellulose membrane, the membranes were blocked with 5% BSA in TBS-T solution followed by overnight incubation at 4°C with primary antibodies against CD142 [TF Antibody (H-9), Santa Cruz Biotechnology, catalog no. sc-374441, RRID:AB_11008609, 1:100], CD252 (Anti-TNFSF4 antibody produced in rabbit, Atlas Antibodies, catalog no. HPA059579, RRID:AB_2684071, 1:1,000), alpha-smooth muscle actin (Anti-alpha smooth muscle Actin antibody, Abcam, catalog no. ab5694, RRID:AB_2223021, 1 μg/mL), or GAPDH [Anti-GAPDH antibody (6C5), Abcam, catalog no. ab8245, RRID:AB_2107448, 1:2,000]. HRP-conjugated secondary antibodies were added at 1:1,000 dilution for 1 hour at room temperature. Imaging was conducted using the ChemiDoc MP Imaging System (Bio-Rad). For phosphorylated protein detection, the same protocol above was used with phospho-FAK (Tyr397l D20B1; Cell Signaling Technology, catalog no. 8556, RRID:AB_10891442), phospho-WNK1 (Thr60; Cell Signaling Technology, catalog no. 4946, RRID:AB_2304531), or phospho-STAT6 (Tyr641) antibody (Cell Signaling Technology, catalog no. 9361, RRID:AB_331595, 1:1,000). For total STAT6, we followed phospho-STAT6 with a 5-minute stripping stage using Restore Western Blot Stripping Buffer (Thermo Fisher Scientific, 21059), before incubation with STAT6 (D3H4) antibody (Cell Signaling Technology, catalog no. 5397, RRID:AB_11220421, 1:1,000).

### Immunofluorescence

Serial sections of a formalin-fixed and paraffin-embedded tumor biopsy with low and high beta-catenin areas were used for immunofluorescence. After rehydration, heat-mediated antigen retrieval was performed by heating the sections in citrate buffer (pH 6.0) in a pressure cooker. The sections were then blocked with 1% BSA + 10% serum (matching secondary antibody host) in PBS-T solution for 1 hour at room temperature. Primary antibodies were applied in blocking solution [1:50 Mouse anti-CD142 (Santa Cruz Biotechnology, sc-374441), 1:200 Rabbit anti-PDPN (Sigma-Aldrich, HPA007534), 1:600 Rabbit anti-CTNNB1 (Sigma-Aldrich, HPA029159)] and the sections were incubated overnight at 4°C. Secondary antibodies were then applied in PBS-T solution containing 1% BSA [1:150 Goat Anti-Mouse Alexa Fluor 568 (Abcam, catalog no. ab175473, RRID:AB_2895153) or 1:100 Goat Anti-Rabbit Alexa Fluor 488 (Abcam, catalog no. ab150077, RRID:AB_2630356)] and the sections were incubated for 1 hour at room temperature. The sections were then mounted with VECTASHIELD Antifade Mounting Medium with DAPI (Vector Laboratories, H-1200) and imaged.

### IHC

Expression of CD142 was evaluated by IHC on tissue microarray TA-349 that contains duplicate cores of 12 desmoid tumor and 12 scar specimens. Tissue microarray sections were deparaffinized, hydrated, boiled for antigen retrieval citrate pH6 buffer, and stained with Anti-Tissue Factor (TF) antibody (EPR8986) at 1:400 dilution (Abcam, catalog no. ab151748, RRID:AB_2814773). Appropriate positive and negative controls were run in parallel. The intensity of the staining was scored as follows: 0 (absent), 1+ (weak), 2+ (distinct), and 3+ (strong).

### Recombinant Protein and STAT6 Inhibitor Treatment

All recombinant proteins were purchased from R&D Systems, reconstituted as per the manufacturer's instructions and used at the following concentrations: Human CCL2 (R&D Systems, 279-MC-010, 100 ng/mL), human CHI3L1 (R&D Systems, 2599-CH-050, 100 ng/mL), human CXCL12 (R&D Systems, 350-NS-010, 100 ng/mL), human IGFBP3 (R&D Systems, 675-B3-025, 100 ng/mL), human PTX3 (R&D Systems, 1826-TS-025, 1 μg/mL), and mouse Wnt3a (R&D Systems, 1324-WN-010, 25 ng/mL). Cells were incubated in serum-free media for 6 hours before treatment with recombinant proteins. Cells were then treated for 18 hours with serum-free media containing the appropriate recombinant protein with or without the STAT6 inhibitor AS 1517499 (Axon Medchem, 1992-10MG, 50 nmol/L). For Wnt3a treatments, 72 hours incubation was used, and media changed with fresh treatment at 24 and 48 hours after initial treatment.

### Statistical Analyses

Statistical calculations were conducted using R (version 4.2.2). k-nearest neighbor distances were calculated using kNNdist function from the *dbscan* package (version 1.1-11). Statistical significance was evaluated using the one- and two-sample *t* tests from base R, with a *P* value less than 0.05 considered significant. The number of experimental replicates and test used is indicated in each figure legend.

### Data Availability

The data generated in this study are available within the article and its Supplementary Data.

## Results

### Desmoid Tumors Contain *CTNNB1* Mutant and Nonmutant Mesenchymal Cells

**FIGURE 1:**
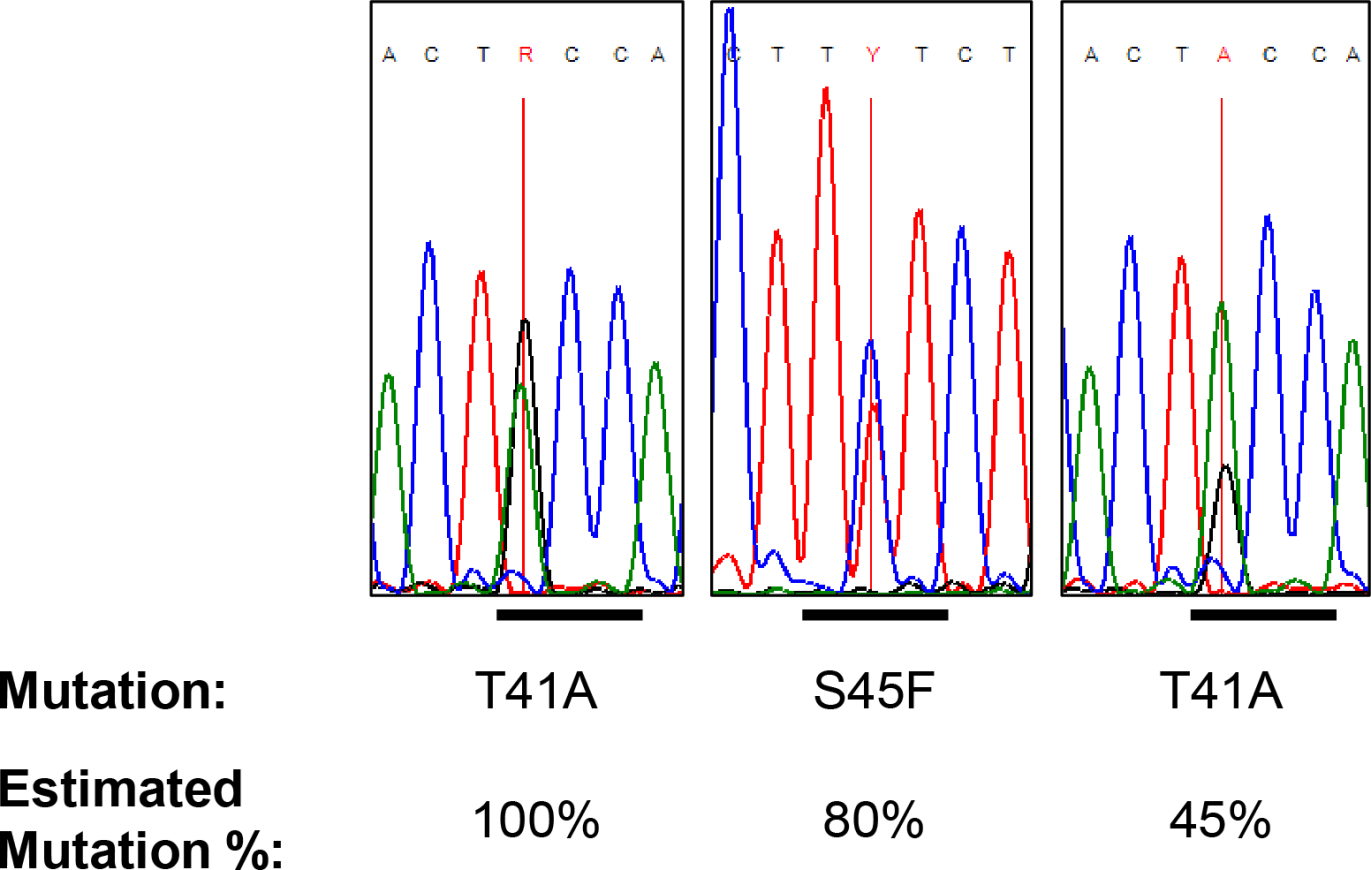
Desmoid tumor primary cultures consist of a variable mixture of mutant and nonmutant populations. Representative Sanger sequencing chromatograms from three samples exhibiting variable mutation frequency. Black horizontal line indicates the altered codon. Red vertical line indicates the point mutation site. Summary of estimated mutation frequency in the full study cohort is reported in Table 1.

To initially characterize the population of beta-catenin (*CTNNB1*) mutant and nonmutant cells in desmoid tumors, we evaluated the mutation status of 15 desmoid tumor primary cultures at their first passage by Sanger sequencing. Nine samples (60%) had a T41A mutation, and four (27%) harbored a S45F mutation. We did not detect *CTNNB1* mutations in two samples (13%). Sanger sequencing chromatograms of most tested samples did not reflect a completely heterozygous population of equal base calls for the unmodified and modified allele, suggesting the presence of heterogeneous populations within each sample. We analyzed the sequencing chromatograms using QSVanalyzer (32) to calculate the relative abundance of the mutant variant to estimate the proportion of mutant cells in each mutant sample (Table 1; Fig. 1). From this analysis, we found that the estimated proportion of mutant cells ranged from approximately 20% to 95% (mutation frequency for all samples was 54% on average, with a standard deviation of 27%; Table 1). There was no appreciable difference in the estimated average mutation frequency between the T41A and S45F samples (49% and 66%, respectively, unpaired *t* test *P* = 0.31). No sample with a detectable mutation displayed more than one mutational event. We also observed that the mutation frequency declines with subsequent passages *in vitro* (Supplementary Fig. S1), suggesting that the current culture conditions favor nonmutant cell growth. Thus, subsequent experiments utilizing primary cultures were conducted on low-passage cultures only (maximum of five passages), and samples were resequenced to confirm their mutational status. Our results demonstrate high variability in the cellular composition of desmoid tumor primary cultures.

**TABLE 1:** Summary of estimated mutation frequency in a collection of 15 desmoid tumor primary cultures

Case ID	Detected *CTNNB1* mutation	Estimated mutant cell frequency	Average frequency per mutation group	Overall average
DT1	T41A	90%	49%	54%
DT2	T41A	83%		
DT3	T41A	75%		
DT4	T41A	53%		
DT5	T41A	41%		
DT6	T41A	30%		
DT7	T41A	24%		
DT8	T41A	24%		
DT9	T41A	20%		
DT10	S45F	95%	66%	
DT11	S45F	80%	(*P* = 0.306)	
DT12	S45F	46%		
DT13	S45F	44%		
DT14	WT	0%		
DT15	WT	0%		

To identify the mutant and nonmutant cells in heterogeneous cultures, we established single cell–derived colonies from two primary cultures (one T41A, and one S45F), both displaying medium (40%–60%) *CTNNB1* mutation frequency. While cells from all colonies displayed a general spindle-like shape, we observed that some colonies exhibited provisionally distinct morphologic and spatial patterns: we observed colonies whose cells tend to appear condensed in morphology and tend to cluster together (we labeled these colonies as Type A), and colonies whose cells display generally more flat morphology and tend to spread apart (labeled as Type B; Supplementary Fig. S2A and S2B). This phenotype was persistent upon subsequent subcloning suggesting a heterogeneity of fibroblastic cell types within the tumor mass. These phenotypic differences resembled those previously reported on fibroblastic monocultures (34, 35). Specifically, our observed Type A colonies resembled fibroblasts while Type B colonies resembled myofibroblasts. Consistent with a myofibroblast-like phenotype, Type B cells expressed higher smooth muscle alpha-actin compared with Type A both at the RNA and protein level (Supplementary Fig. S3).

**FIGURE 2:**
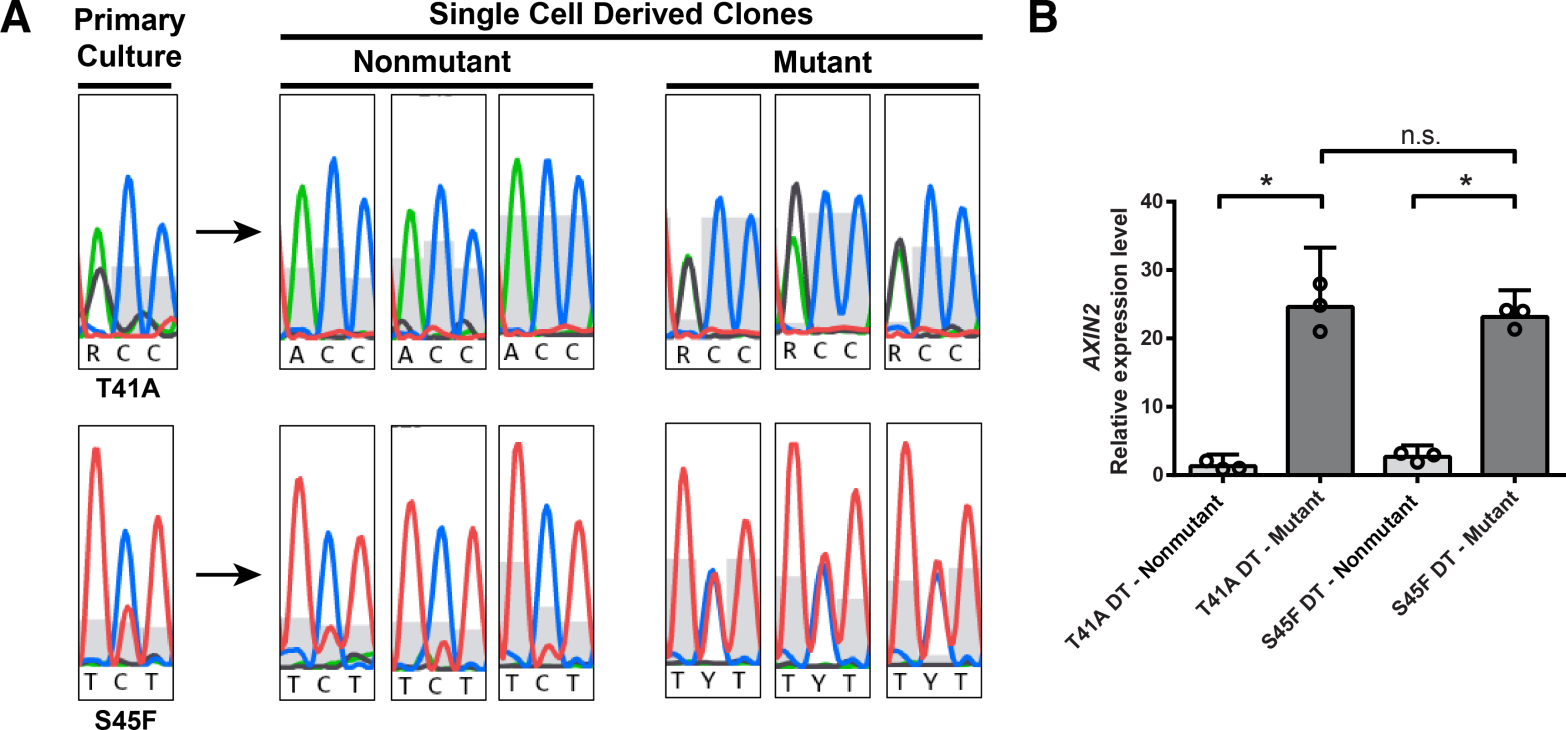
Single cell–derived clonal expansion of desmoid tumor samples isolates mutant and nonmutant populations. **A,** Sanger sequencing chromatograms for desmoid tumor samples with a *CTNNB1* T41A and S45F mutations. The sequence of the original primary culture is shown on the left and chromatograms of six colonies grouped by their mutation status are shown on the right. **B,***AXIN2* expression was measured by real-time quantitative PCR as an indicator of beta-catenin transcriptional activity. Colonies were grouped by the mutation of their original primary culture (T41A or S45F) and separated by their actual *CTNNB1* sequencing result (mutant or nonmutant). Data represent mean + 95% confidence interval. * Sidak multiple comparisons test *P* < 0.05 (*n* = 3 colonies per sample, n.s. = “not significant”). Circles indicate individual datapoints.

We then analyzed at least three colonies for each subphenotype by Sanger sequencing of *CTNNB1*. Peak height analysis of sequencing chromatograms supported the clonality of each subpopulation derived using this approach. Type A colonies with clustered growth showed no detectable mutations in *CTNNB1*, while Type B colonies with dispersed growth carried the mutation detected in their original primary culture (Fig. 2A). Because the detected beta-catenin mutations are known to increase its transcriptional activity (4, 31), we analyzed the gene expression of *AXIN2*, a known transcriptional target of beta-catenin (36). As expected, the mutant colonies exhibited elevated *AXIN2* expression compared with nonmutant colonies (Fig. 2B). We detected no difference in *AXIN2* expression between T41A and S45F mutant colonies. We also established additional single cell–derived colonies from two high (>80%) and three low (<40%) mutational frequency primary cultures but we these colonies did not exhibit distinct morphologic or genotype differences. Thus, our clonal expansion method can isolate mutant and nonmutant subpopulations from heterogeneous primary desmoid tumor cultures of medium mutational frequency. This likely requires increasing the number of colonies generated and selected per sample to improve the detection power of mutant colonies in samples with low tumor fraction. Larger-scale experiments are needed to investigate the relationship between mutation status and morphology.

### High-throughput Cell Surface Profiling Distinguished Mutant from Nonmutant Colonies in Desmoid Tumors

Proteins anchored to the cell membrane can be used as relatively accessible markers to distinguish between cell populations, but such proteins have not been identified in mesenchymal tumors. We performed a high-throughput surface antigen screen on two desmoid tumor samples (one T41A and one S45F), as described previously (26, 33), to evaluate the expression of 368 surface antigens by flow cytometry to identify such markers (Supplementary Table S1). In both samples, we profiled *CTNNB1* mutant colonies and *CTNNB1* wildtype colonies derived from the same desmoid sample, for a total of four screens. In general, we observed a very similar surface protein expression profile between mutant and wildtype colonies. This profile was consistent with previously reported fibroblast surface proteome. We found 24 proteins that were present in more than 50% of cells within all tested colonies (whether *CTNNB1* mutant or not). This class of proteins included known fibroblastic markers such as CD13, CD29, CD44, and CD73 (37–39). When we compared our results with a previously published cell surface profile of normal dermal fibroblasts using the same flow cytometry–based screen (26), we found 21 (88%) of the 24 expressed proteins were also positive in dermal fibroblasts; the remaining three proteins were still detectable in more than 10% of normal dermal fibroblasts. Conversely, 290 proteins were detected in less than 50% of cells in all tested subgroups in our study. Of these, we found 278 (96%) proteins were also negative in the dermal fibroblast profile; four proteins in our screen were expressed in more than 50% of normal dermal fibroblasts (CD21, CD66e, CD104, and CD151), and the remaining nine were not tested in the previously published profile. Our results are therefore comparable with previously published work and provide additional evidence for the reproducibility of this screen despite the small number of biological replicates. Given the fibroblastic nature of desmoid cells, we expectedly found that mutant and nonmutant colonies shared a very similar fibroblastic expression pattern for most proteins probed in this study. For example, five proteins (CD49E, CD90, CD73, CD44, and CD13) were previously identified as candidate cancer-associated fibroblast markers in serous ovarian cancer using this screening strategy (26), but here we found all proteins were expressed by all subpopulations at greater than 80% positivity (Supplementary Table S1), supporting the need to profile distinct subpopulations within mesenchymal tumors.

**FIGURE 3:**
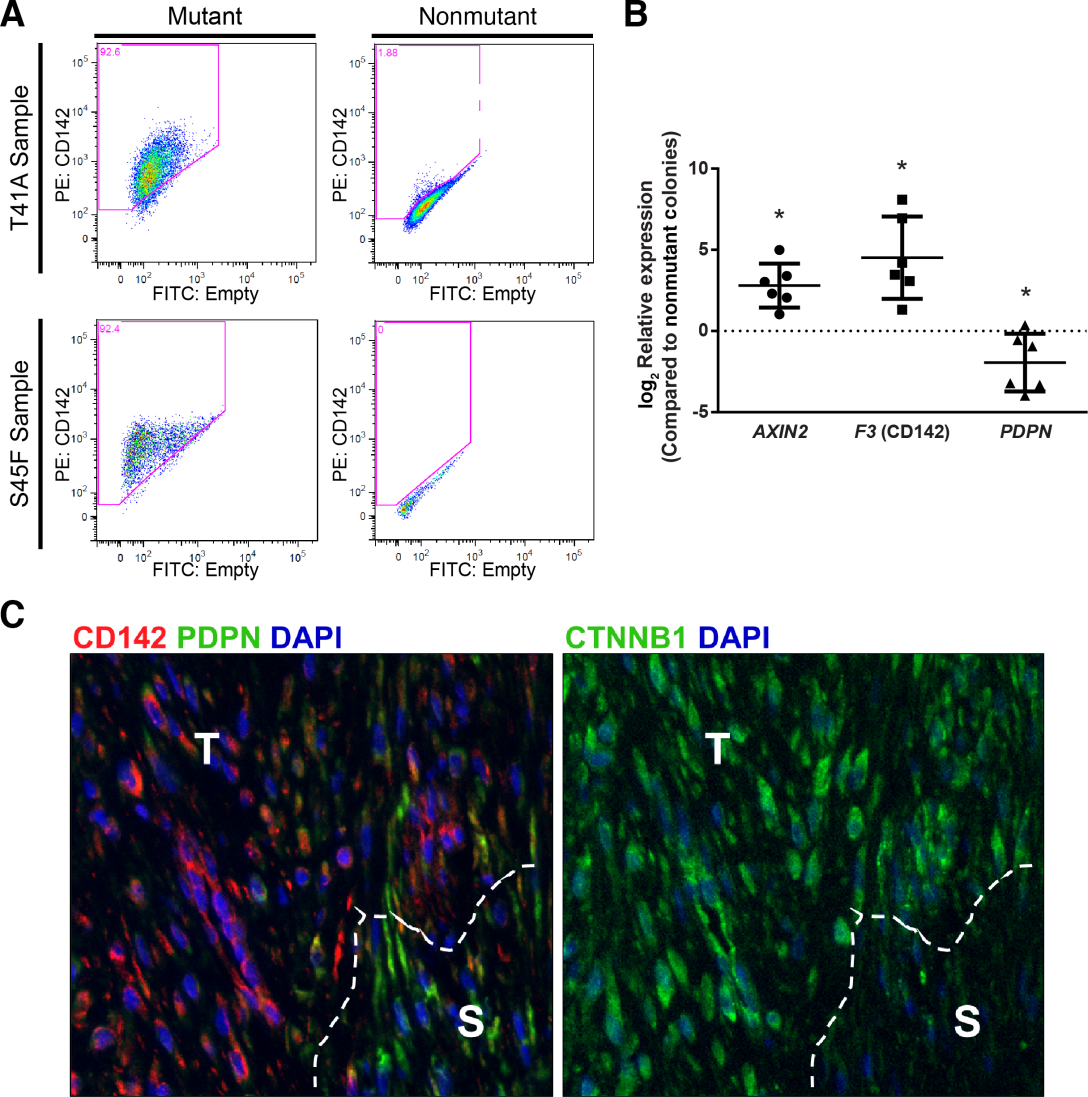
Clonal cell surface profiling identifies CD142 as a potential marker of mutant cells in heterogeneous desmoid tumor samples. **A,** Flow cytometry analysis of two desmoid tumor samples (one carrying the T41A mutation, and one carrying S45F) after isolating into mutant and nonmutant colonies by clonal expansion. CD142 was identified as enriched in the mutant colonies of both samples (92.6% and 92.4%, respectively) but not in their nonmutant colonies (1.88% and 0%). The *x*-axis (FITC) is an empty channel as no FITC-conjugated antibodies were used in this experiment. **B,** Quantitative PCR analysis of mutant and nonmutant colonies in desmoid tumor samples showing increased *AXIN2* and *F3* (which encodes CD142) and decreased podoplanin (*PDPN*) in mutant colonies (*n* = 6 samples, * one-sample *t* test *P* < 0.05). **C,** Immunofluorescence staining of CD142, PDPN and beta-catenin in a desmoid tumor sample (T: Tumor, S: Stroma, as identified by visually inspecting beta-catenin staining intensity).

Despite the similarity in the cell surface profiles, few surface proteins tested were expressed primarily by one subgroup (Supplementary Table S2). Of these, CD142 (also known as tissue factor, TF, encoded by *F3*) was identified as a candidate positive marker of mutant cells in both experiments (Fig. 3A). We also identified podoplanin as a marker enriched in the nonmutant population (Supplementary Fig. S4). To validate the results, we compared the relative gene expression level of CD142 (encoded by *F3*) and podoplanin in a larger set of mutant and nonmutant colonies derived from six samples. We observed that the mutant populations have higher expression of *F3* relative to nonmutant populations derived from the same desmoid tumor samples (*n* = 6, *P* < 0.05; Fig. 3B). Conversely, podoplanin expression is lower in the mutant colonies. We next aimed to study the expression of our candidate surface marker *in situ.* First, we performed double immunofluorescent staining for CD142 and PDPN in a human desmoid tumor biopsy showing high and low beta-catenin staining regions (Fig. 3C). We observed that CD142 staining matched beta-catenin staining pattern. Conversely, podoplanin staining was higher in low beta-catenin regions. To determine whether CD142 is expressed in a broader range of desmoids, we performed IHC staining on a tissue microarray assembled from 12 desmoid tumor patient samples. Positive staining for CD142 was observed in all 12 cases (Table 2). We then examined dermal scar tissues, since we have previously shown that beta-catenin is stabilized in these tissues (29, 40), and detected positive CD142 staining in a panel of 10 core biopsies from scar tissues (Supplementary Table S3; Supplementary Fig. S11). Thus, CD142 overexpression may be a marker of beta-catenin activation, and not restricted to *CTNNB1* mutational status. Treatment of wildtype dermal fibroblasts with recombinant Wnt3a showed a trend toward increased CD142 expression, encoded by *F3,* but the differences were not statistically significant (Supplementary Fig. S5). Taken together, our data identify CD142 and podoplanin as potential surface markers that are differentially expressed between cells with mutant, active beta-catenin and nonmutant, inactive beta-catenin in heterogeneous desmoid tumor samples.

**TABLE 2:** IHC analysis of CD142 in a desmoid tumor tissue microarray. Results from duplicate cores per case are shown when available

Case ID	Positive cells (%)	Stain intensity
6421	90	2+
	0	0
6264	90	3+
	100	3+
22606	80	1+
	90	2+
22607	95	3+
	95	3+
22608	95	3+
	95	3+
22609	80	3+
	90	3+
22610	90	3+
	90	3+
22611	70	3+
	70	3+
22612	70	2+
22613	30	2+
	50	2+
22614	100	3+
	100	3+
22615	100	3+
	100	3+

### CD142 Identifies the Mutant Cell Population in Desmoid Tumors

**FIGURE 4:**
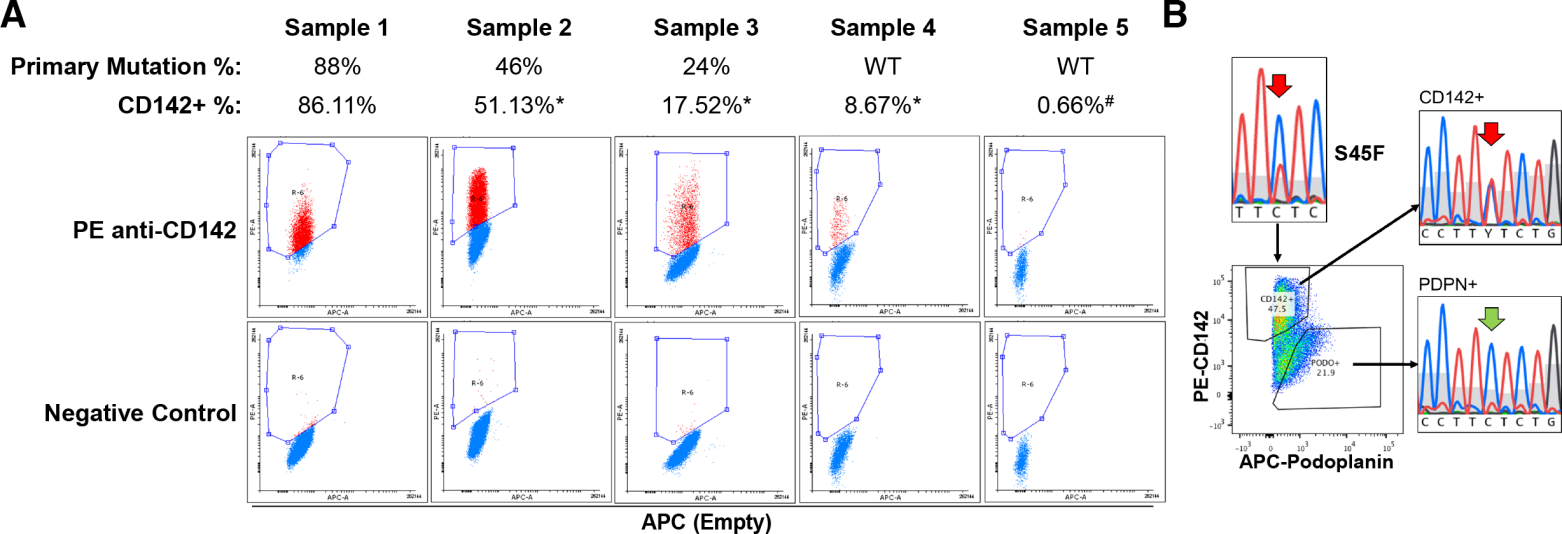
Isolating the mutant population from heterogeneous desmoid tumors by CD142-based sorting. **A,** Flow cytometry analysis of five primary desmoid tumor cultures shows that the proportion of CD142^+^ cells correlates with estimated mutation frequency. * indicates samples whose CD142^+^ cells were subsequently isolated by FACS and confirmed to be mutant by Sanger sequencing. # indicates CD142^+^ cells were sorted but could not be expanded *in vitro*. Pearson correlation coefficient *r* = 0.9874 (*P* = 0.0017) of CD142^+^ to estimated mutation frequency. The *x*-axis (APC) is an empty channel as no APC-conjugated antibodies were used in this experiment. **B,** Schematic of double-color cell sorting using CD142 and podoplanin showing a chromatogram of the original sample and its isolated CD142^High^;PDPN^Low^ (CD142^+^) and CD142^Low^;PDPN^High^ (PDPN+) subpopulations.

To investigate the utility of CD142 to isolate mutant cells from heterogeneous primary desmoid tumor cultures, we conducted CD142-based flow cytometry and FACS of three primary cultures with a known mutation and the two primary cultures in which we did not detect any mutations in *CTNNB1* using Sanger sequencing. Analysis of these samples shows the proportion of CD142-positive (CD142^+^) cells correlated with the estimated mutation frequency from Sanger sequencing experiments conducted in parallel (Pearson correlation coefficient *r* = 0.9874, *P* = 0.0017; Fig. 4A; Supplementary Fig. S6). We then expanded the sorted CD142^+^ cells and analyzed them by Sanger sequencing. In the three primary cultures with previously known mutation, the CD142^+^ cells were enriched (>95% estimated mutation frequency) for the same *CTNNB1* mutation. Notably, 8.67% of the cells in one of the two primary cultures previously labeled as “wildtype” were also labeled as CD142^+^. Subsequent expansion and sequencing of these cells revealed an S45F mutation at approximately 50% mutation frequency. Our detection of a minor mutant subpopulation in one of the samples that failed mutation detection by Sanger is consistent with findings that traditional sequencing methods of analyzing bulk tumors are not sensitive enough to detect a small population of mutant cells (5, 6). We then studied the utility of multicolor staining in our FACS experiments. In CD142/PDPN double-staining experiments, we detected both CD142^High^;PDPN^Low^ and CD142^Low^;PDPN^High^ populations which corresponded to subpopulations enriched for mutant and nonmutant *CTNNB1*, respectively (Fig. 4B; Supplementary Table S4). FACS isolated mutant and nonmutant subpopulations displayed similar morphologic differences in monoculture (Supplementary Fig. S7) as was previously observed when comparing single cell–derived mutant and nonmutant colonies. Overall, CD142-based FACS offers an alternative method to enrich for the mutant population in heterogeneous desmoid tumor primary cultures.

### Stroma-derived Secreted Factors Induce Mutant Cell Proliferation

**FIGURE 5:**
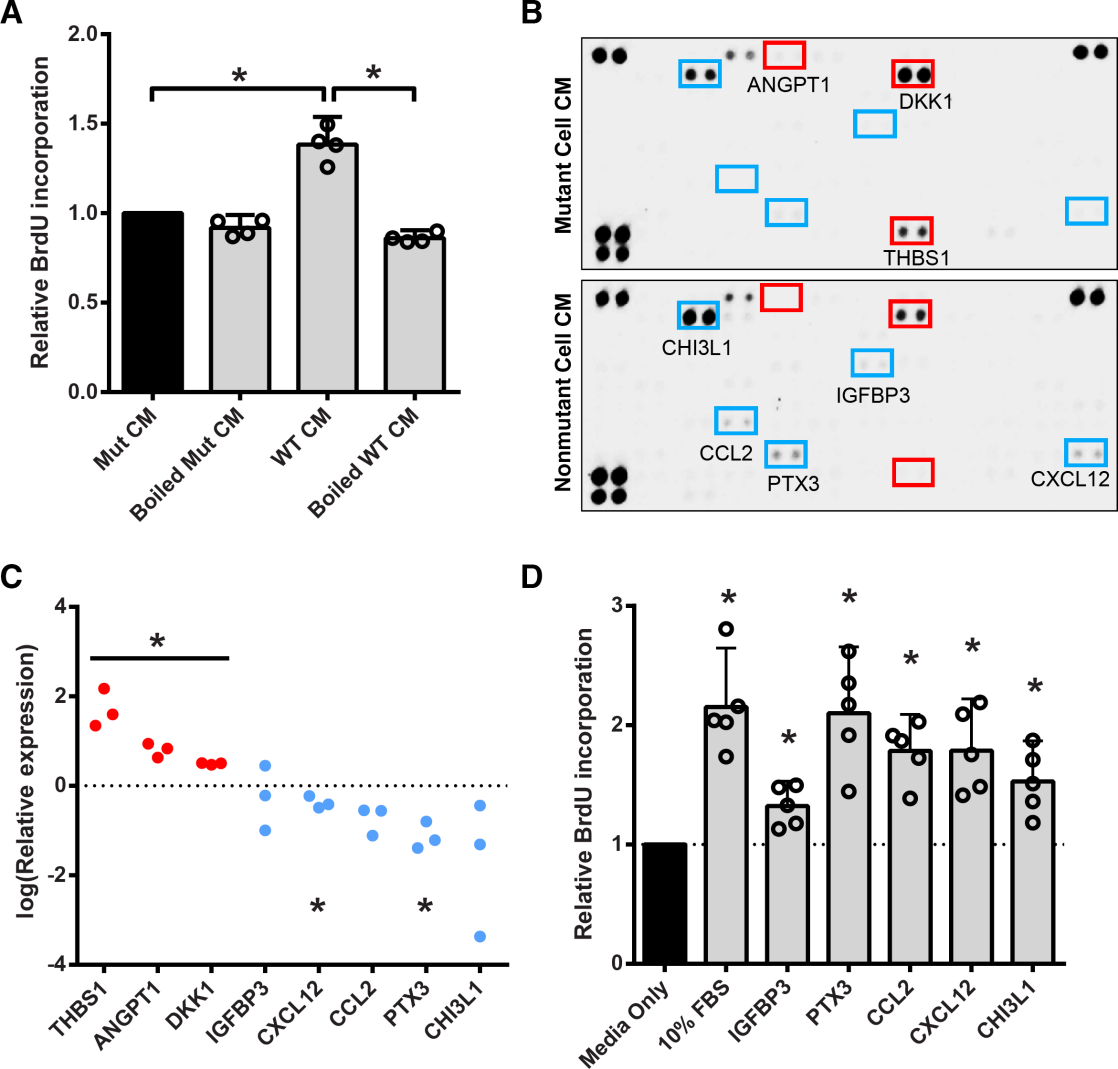
Nonmutant fibroblasts secrete soluble factors that induce mutant desmoid tumor cell proliferation. **A,** Exposure of mutant cells to nonmutant conditioned media increases their proliferation compared with treatment with mutant conditioned media. This effect was abrogated upon heat inactivation. Repeated measures ANOVA *P* < 0.0001. * Sidak multiple comparisons test *P* < 0.05 (*n* = 4). **B,** Representative image of a cytokine antibody array assay comparing conditioned media from mutant (top) and nonmutant (bottom) cells derived from the same desmoid tumor sample. Duplicates in boxes are cytokines that displayed consistent pattern in another biological replicate (see Supplementary Fig. S7 and S8). **C,** Gene expression of candidate cytokines was measured by quantitative PCR (*n* = 3, * one sample *t* test *P* < 0.05). **D,** Treatment of mutant cells with recombinant secreted factors in serum-free media increases their BrdU incorporation compared with media only. Repeated measures ANOVA *P* < 0.0001. * Dunnett multiple comparisons test *P* < 0.05 (*n* = 5). Data are represented as mean + 95% confidence intervals. Circles indicate individual datapoints.

Stroma-derived soluble factors that function in paracrine signaling can alter tumor cell behavior. To determine whether paracrine signaling is active in desmoid tumors, we cocultured mutant desmoid tumor cells with nonmutant fibroblasts using permeable inserts to allow soluble factors to be shared between the two populations without direct cell contact. Compared with coculturing with other mutant cells, mutant cells cocultured with nonmutant fibroblasts exhibited a higher proliferation rate as measured by BrdU incorporation (Supplementary Fig. S8). To determine whether nonmutant cell-derived cytokines are necessary for the increased proliferation, we treated isolated mutant cells with nonmutant cell conditioned media with and without heat denaturation. Consistent with our coculture experiment, conditioned media derived from nonmutant cells increased the proliferation rate of mutant desmoid tumor cells compared with conditioned media derived from other mutant cells. Notably, this effect was abrogated upon heat inactivation (Fig. 5A). To determine which secreted factors are differentially produced by nonmutant cells, we analyzed conditioned media from mutant and nonmutant cell populations by a cytokine antibody array. We found DKK1, THBS1, and ANGPT1 are elevated in the conditioned media of mutant cells while IGFBP3, PTX3, CXCL12, CCL2, and CHI3L1 were elevated in the conditioned media of nonmutant cells (Fig. 5B; Supplementary Figs. S9 and S10). Transcripts of these identified secreted factors were similarly differentially regulated as measured by quantitative real-time PCR in an independent set of samples (Fig. 5C). To identify candidate stroma-derived factors that might influence desmoid tumor cell proliferation, we treated mutant-only cultures with recombinant IGFBP3, PTX3, CCL2, CXCL12, and CHI3L1 in serum-free conditions. We observed that PTX3, CCL2, and CXCL12 significantly increased BrdU incorporation compared with no treatment (Fig. 5D). Therefore, our data demonstrate differential expression of secreted factors in mutant and nonmutant cells in desmoid tumors. Moreover, nonmutant fibroblasts have the potential to increase the proliferation of mutant desmoid tumor cells by secreting soluble factors that include PTX3, CCL2, and CXCL12.

### The STAT6 Inhibitor AS1517499 Blocks Stroma-induced Proliferation of Mutant Cells

**FIGURE 6:**
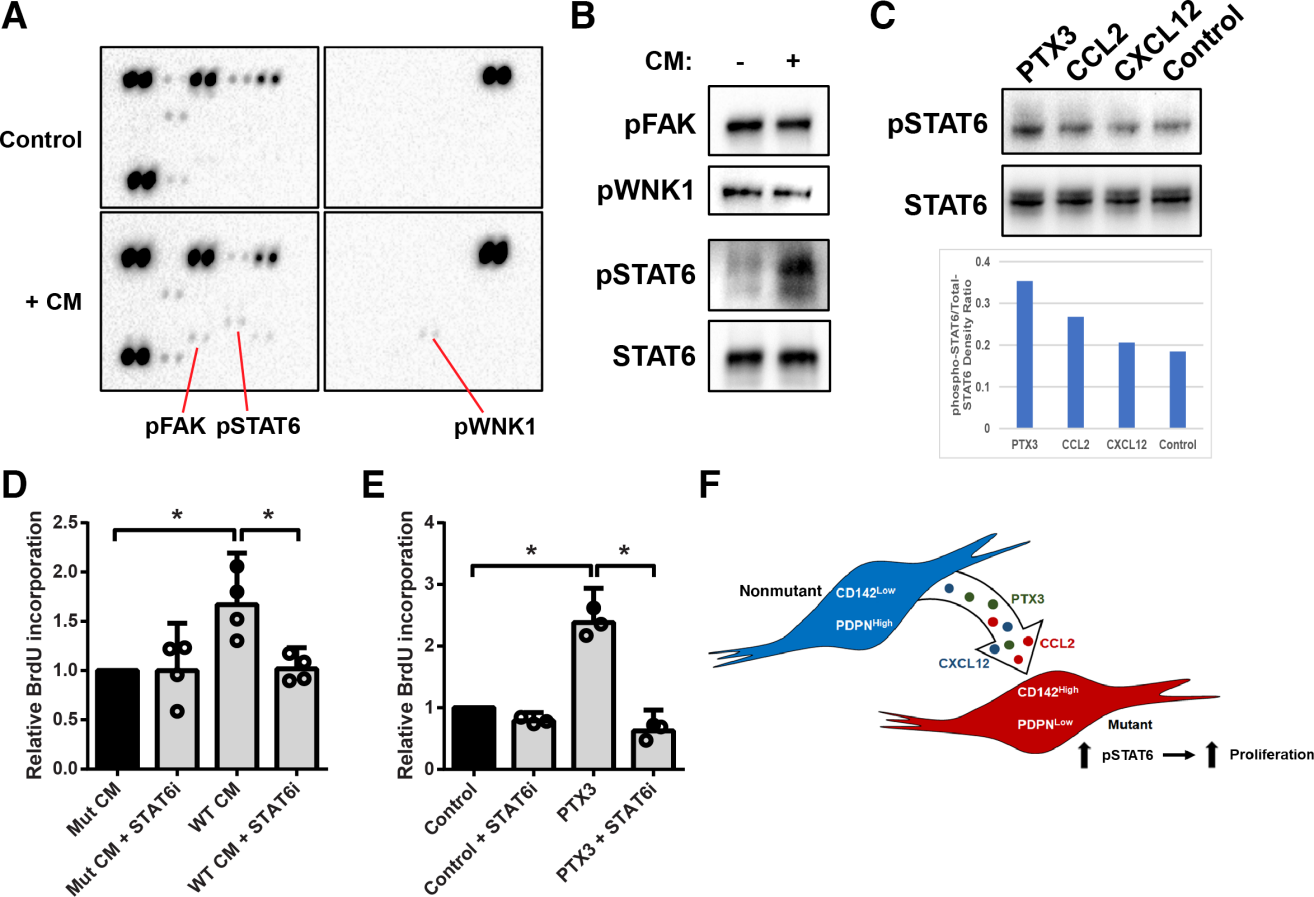
Nonmutant soluble factors phosphorylate STAT6 in mutant desmoid tumor cells. **A,** Representative phosphoproteome antibody array after treatment of mutant cells with nonmutant conditioned media. Control is exposure to mutant conditioned media. **B,** Immunoblot verification of candidate phosphoproteins. **C,** Treatment of mutant cells with recombinant soluble factors increases phospho-STAT6 level (representative immunoblot of two experiments). **D,** Treatment of mutant cells with nonmutant conditioned media with the STAT6 inhibitor AS1517499 (STAT6i) blocks the proliferation increase. Repeated measures ANOVA *P* = 0.0023, * Sidak multiple comparisons test *P* < 0.05 (*n* = 4). **E,** AS1517499 also blocks the proliferation increase after treatment with recombinant PTX3. Repeated measures ANOVA *P* < 0.0001. * Sidak multiple comparisons test *P* < 0.05 (*n* = 3). **F,** A schematic representing our current model: Nonmutant stromal fibroblasts (with high podoplanin and low CD142 surface marker expression) secrete PTX3, CCL2, and CXCL12, among other soluble factors, which phosphorylates STAT6 leading to enhanced proliferation in desmoid tumor cells carrying a *CTNNB1* mutation (with high CD142 and low podoplanin expression). Data are represented as mean + 95% confidence intervals. Circles indicate individual datapoints.

To investigate the pathways activated in by the conditioned media, we used a phosphokinase antibody array to analyze the mutant cells treated with nonmutant conditioned media and verified results by Western blotting. We detected increased phosphorylation of STAT6 following conditioned media treatment (Fig. 6A and B). To determine whether the identified stroma-derived secreted factors contributed to STAT6 phosphorylation, we treated mutant desmoid tumor cells with recombinant secreted factors differentially expressed by the nonmutant cells and found that PTX3 induced the highest increase in phospho-STAT6 (Fig. 6C). To test whether STAT6 activity influenced mutant cell proliferation, we used AS1517499, a potent STAT6 inhibitor (41). Treating mutant cells with nonmutant conditioned media in combination with 50 nmol/L of AS1517499 abrogated the effect of increased proliferation from treating with conditioned media alone (Fig. 6D). To determine whether PTX3 induces desmoid tumor cell proliferation via STAT6 activation, we combined recombinant PTX3 protein treatment with AS1517499. We found that while PTX3 treatment alone increased BrdU incorporation as we found previously, STAT6 inhibition by AS1517499 abrogated this increase (Fig. 6E). Taken together, our data suggest that nonmutant stromal fibroblasts induce mutant desmoid tumor cell proliferation via secreting soluble factors, including PTX3 and STAT6 activation (Fig. 6F).

## Discussion

Here we describe the isolation of mutant and nonmutant subpopulations from heterogeneous desmoid tumor samples by clonal expansion and surface marker–based cell sorting. Our study identifies CD142 as a marker enriching for the mutant population which can be used to isolate the mutant population using conventional cell sorting methods for downstream profiling. Furthermore, we found that nonmutant cells can contribute to mutant cell proliferation via secreted factors. PTX3, CCL2, and CXCL12 are stroma-derived factors that increase the proliferation rate of mutant desmoid tumor cells. STAT6 partially mediates the ability of stromal cells to induce proliferation in mutant cells.

The mutation status of desmoid tumors has traditionally been determined by Sanger sequencing and not all tumors had mutations identified. Deep sequencing to identify minor mutant populations within heterogeneous samples (5) showed a higher mutation rate. The proportion of mutant and nonmutant cells within the tumor may contribute to differences in mutation detection rates, especially when Sanger sequencing is utilized. Interestingly, there has been a suggestion that nonmutant tumors have a better prognosis (42–44). It is possible that the “wildtype” tumors in these studies had beta-catenin mutations in a small minority of tumor cells, and instead the better prognosis in “wildtype” tumors could be related to a lower mutant tumor cell burden. An analysis of the proportion of CD142^+^ cells in desmoid tumors and prognosis would be an interesting clinical study.

CD142, also called TF, is a member of the blood coagulation cascade, but recent evidence suggests it may also be involved in additional cellular signaling important for tumor growth (45). Structurally, CD142/TF shares an evolutionary lineage with the type II cytokine receptor family which also includes the IFN receptors and IL receptors. Mechanistically, CD142/TF can activate a signaling pathway by its interaction with FVIIa which has been shown to lead to progrowth ERK1/2 phosphorylation. These mechanisms have been implicated in angiogenesis (46) and metastasis (47) in melanoma models. Data from the Human Protein Atlas (www.proteinatlas.org) show that high TF expression is associated with unfavorable survival outcome in renal and pancreatic cancers. These findings raise the possibility that CD142 may play a functional role in maintaining desmoid tumor growth. Given its aberrant expression in other solid tumors and its known internalizing capacity (48), an antibody–drug conjugate targeting CD142 has also been studied to induce tumor regression in patient-derived xenograft models of solid tumors (49) and a recent multicenter phase I–II trial demonstrated its manageable safety profile in patients with solid tumor (NCT02001623; ref. 50). Given that CD142 is enriched in desmoid tumor *CTNNB1* mutant cells, targeting CD142 in desmoid tumors for cytotoxic drug delivery may offer a promising therapeutic option.

Our study has some limitations. It is possible that surface protein expression is influenced by the specific *CTNNB1* variant. Given the small number of samples in our study, however, we cannot distinguish whether the higher general expression of markers (e.g., CD120A, CD123, CD178) in cells derived from T41A mutant tumors thought to be a result of tumor biology with differences in both tumor and stroma resulting from that specific mutation and subsequent signaling alterations, or artifact of the two experiments being performed separately. We therefore focused our study on overlapping markers, a criterion only CD142 met in this study. Future experiments that are conducted at a larger scale can help distinguish whether specific *CTNNB1* variants yield a difference in tumor and stroma surface protein expression. It is also possible that additional proteins found in a minority of our cells could still be useful to identify subpopulations which may harbor important biological functions. Moreover, our study is limited by the antibodies included in the high-throughput flow cytometry panel used. As such, the utility of additional surface proteins such as FAP, which was not included in this panel, could not be determined. Given that the screen was conducted on cultured cells, it is also possible that *in vitro* culture conditions impacted the expression pattern of some markers.

Our study demonstrates that STAT6-activating secreted factors contribute to mutant cell proliferation. Evidence from other cancer types suggests a role for STAT6 activation in tumor progression. High STAT6 expression correlates with worse prognosis in colorectal cancer (51) and prostate cancer (52). STAT6 is also often found constitutively phosphorylated in Hodgkin and Reed-Sternberg cells of Hodgkin lymphoma (53). Treatment with AS1517499 was previously shown to reduce tumor growth and early liver metastasis in an orthotopic 4T1 mammary carcinoma mouse model (54), suggesting that such a strategy may also be promising for desmoid tumors.

To date, the investigation of the role of stromal cells in mesenchymal tumors is hampered by difficulties distinguishing them from the neoplastic cells. Our study shows that lineage-specific surface markers would perform poorly in distinguishing functionally and molecularly distinct cell subpopulations in a mesenchymal tumor. Here, we identified surface markers to quantify and isolate mutant and nonmutant cell subpopulations in desmoid tumors, and to study their interactions via soluble factors. Despite the relative uniformity in beta-catenin aberrations across the majority of patients with desmoid tumor, the disease exhibits a diversity of clinical behavior. Because our study demonstrates the cross-talk between subpopulations, it is possible that the proportions of mutant and nonmutant subpopulations in each tumor, and their respective secretome, may be an important factor that determines disease progression and response to therapy, which warrants further investigation. We also anticipate our clonal expansion and profiling strategy to be useful to study tumor–stroma interactions in other sarcomas. In desmoid tumors, *CTNNB1* hotspot mutations can be sequenced with a single primer pair, but other techniques such as multiplexed amplicon sequencing, hybridization capture panels, or whole-exome/whole-genome sequencing can be deployed in diseases where there is no single disease-defining mutation.

## Supplementary Material

Supplementary Figure S1 Decreasing mutational frequency with increasing passages in a desmoid tumor primary culture.

Supplementary Figure S2 Detection of potential morphological differences between colonies derived from the same desmoid tumor primary culture sample

Supplementary Figure S3 Type B (mutant) cells exhibit higher alpha-smooth muscle actin levels at the protein and RNA level.

Supplementary Figure S4 Podoplanin-based flow cytometry of mutant and non-mutant colonies.

Supplementary Figure S5 Measuring gene expression of F3 and PDPN after Wnt3a treatment of skin fibroblasts

Supplementary Figure S6 Correlation between estimated mutation frequency from Sanger sequencing and percent CD142-positive cells in heterogeneous desmoid tumor samples

Supplementary Figure S7 Morphological differences in mutant and non-mutant cells isolated by FACS sorting

Supplementary Figure S8 Co-culturing of mutant desmoid tumor cells with non-mutant fibroblasts increases their proliferation rate

Supplementary Figure S9 Cytokine antibody array to identify differentially secreted factors from isolated mutant and non-mutant cells.

Supplementary Figure S10 Densitometry of selected secreted proteome from mutant and non-mutant conditioned media

Supplementary Figure S11 Histologic appearance of two cases of desmoid tumors with different patterns of CD142 expression

Supplementary Figure S12 Representative gating strategy

Supplementary Table S1 Cell surface profiling data of mutant and non-mutant desmoid tumor-derived colonies. Name of the sample indicates the mutation status (mutant (MUT) or wildtype (WT)) of the pooled colonies and the genotype (T41A or S45F) of the original primary sample.

Supplementary Table S2 Enriched markers in mutant and non-mutant colonies from our high throughput surface antigen screen.

Supplementary Table S3 CD142 immunohistochemical analysis in scar tissue cores. Results from duplicate cores per do are shown when available.

Supplementary Table S4 Summary of double-color cell sorting experiments.

Supplementary Table S5 Primers used in real-time quantitative PCR experiments.
