# Supplementary material for: CD142 Identifies Neoplastic Desmoid Tumor Cells, Uncovering Interactions Between Neoplastic and Stromal Cells That Drive Proliferation
Source: Cancer Res Commun. 2023 Apr 25;3(4):697–708. doi: 10.1158/2767-9764.CRC-22-0403 (PMC10128091; doi:10.1158/2767-9764.CRC-22-0403)
Supplement: Supplementary Figure S1 — Decreasing mutational frequency with increasing passages in a desmoid tumor primary culture. [file crc-22-0403-s01.docx]

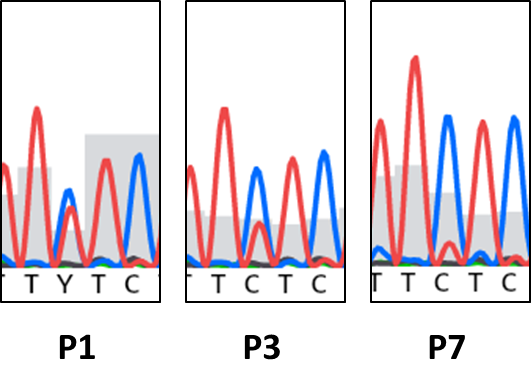


Supplementary Figure S1. Decreasing mutational frequency with increasing passages in a desmoid tumor primary culture.

Passage number is indicated below each chromatogram. Estimated mutation frequency is 80% (P1), 45% (P3), and <10% (considered “wildtype”) (P7).
