# Supplementary material for: CD142 Identifies Neoplastic Desmoid Tumor Cells, Uncovering Interactions Between Neoplastic and Stromal Cells That Drive Proliferation
Source: Cancer Res Commun. 2023 Apr 25;3(4):697–708. doi: 10.1158/2767-9764.CRC-22-0403 (PMC10128091; doi:10.1158/2767-9764.CRC-22-0403)
Supplement: Supplementary Figure S2 — Detection of potential morphological differences between colonies derived from the same desmoid tumor primary culture sample [file crc-22-0403-s02.docx]

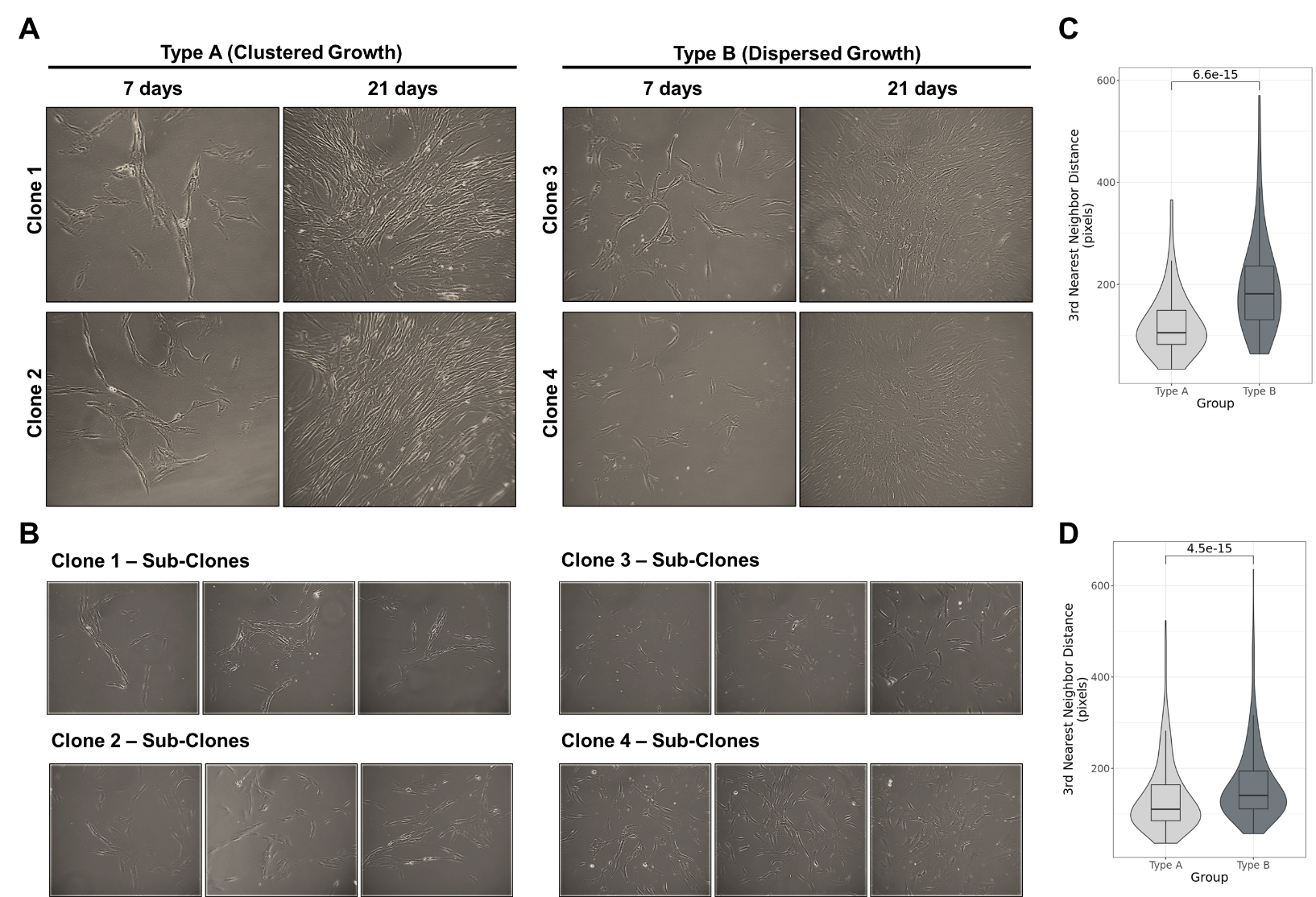


Supplementary Figure S2. Detection of potential morphological differences between colonies derived from the same desmoid tumor primary culture sample.

**(A)** Representative micrographs of colonies derived from the same desmoid tumor primary culture displaying a clustered (Type A) growth pattern or dispersed (Type B) pattern. **(B)** These morphological differences appear to have persisted after subcloning. **(C)** Distance measured between any cell and its 3^rd^ nearest neighbor was higher in Type B colonies compared to Type A. **(D)** This difference persisted after subcloning. P values of t-test shown in both (C) and (D).
