# Supplementary material for: CD142 Identifies Neoplastic Desmoid Tumor Cells, Uncovering Interactions Between Neoplastic and Stromal Cells That Drive Proliferation
Source: Cancer Res Commun. 2023 Apr 25;3(4):697–708. doi: 10.1158/2767-9764.CRC-22-0403 (PMC10128091; doi:10.1158/2767-9764.CRC-22-0403)
Supplement: Supplementary Figure S3 — Type B (mutant) cells exhibit higher alpha-smooth muscle actin levels at the protein and RNA level. [file crc-22-0403-s03.docx]

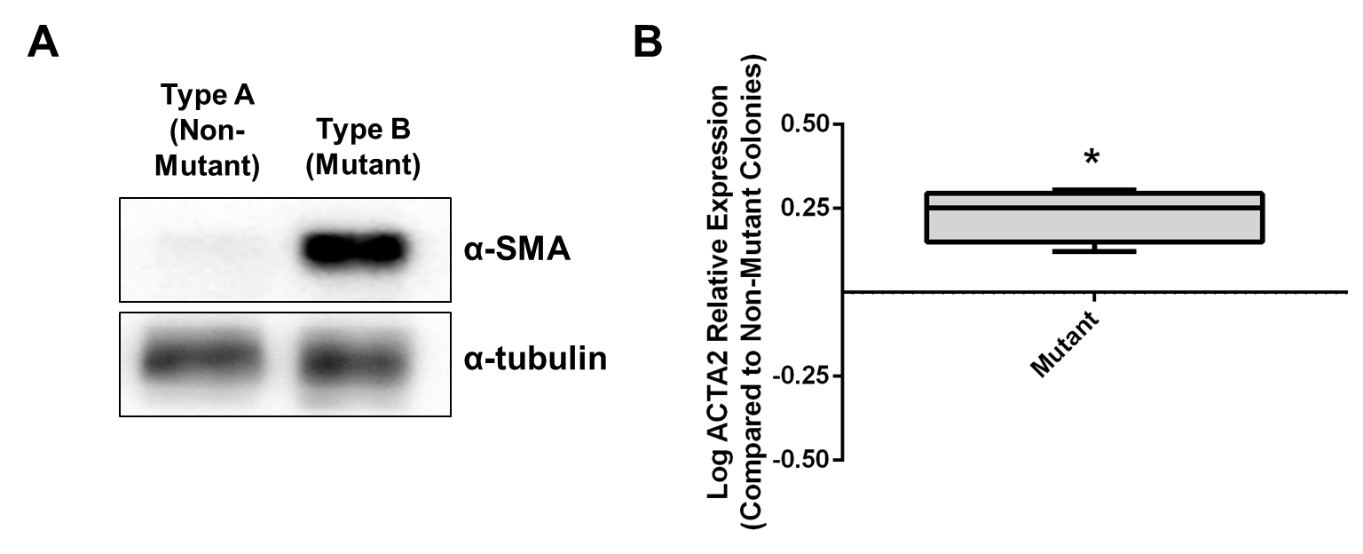


Supplementary Figure S3. Type B (mutant) cells exhibit higher alpha-smooth muscle actin levels at the protein and RNA level. (A) An immunoblot of alpha-smooth muscle actin protein showing detection of a-SMA in Type B (*CTNNB1* mutant) cultures but not in Type A (*CTNNB1* wildtype) cultures. (B) Real-time quantitative PCR was used to compare *ACTA2* expression between *CTNNB1-*mutant and -wildtype cultures derived from the same sample. *** one-sample t-test P < 0.05 (n=4).
