# Supplementary figures and images for: CD142 Identifies Neoplastic Desmoid Tumor Cells, Uncovering Interactions Between Neoplastic and Stromal Cells That Drive Proliferation
Source: Cancer Res Commun. 2023 Apr 25;3(4):697–708. doi: 10.1158/2767-9764.CRC-22-0403 (PMC10128091; doi:10.1158/2767-9764.CRC-22-0403)

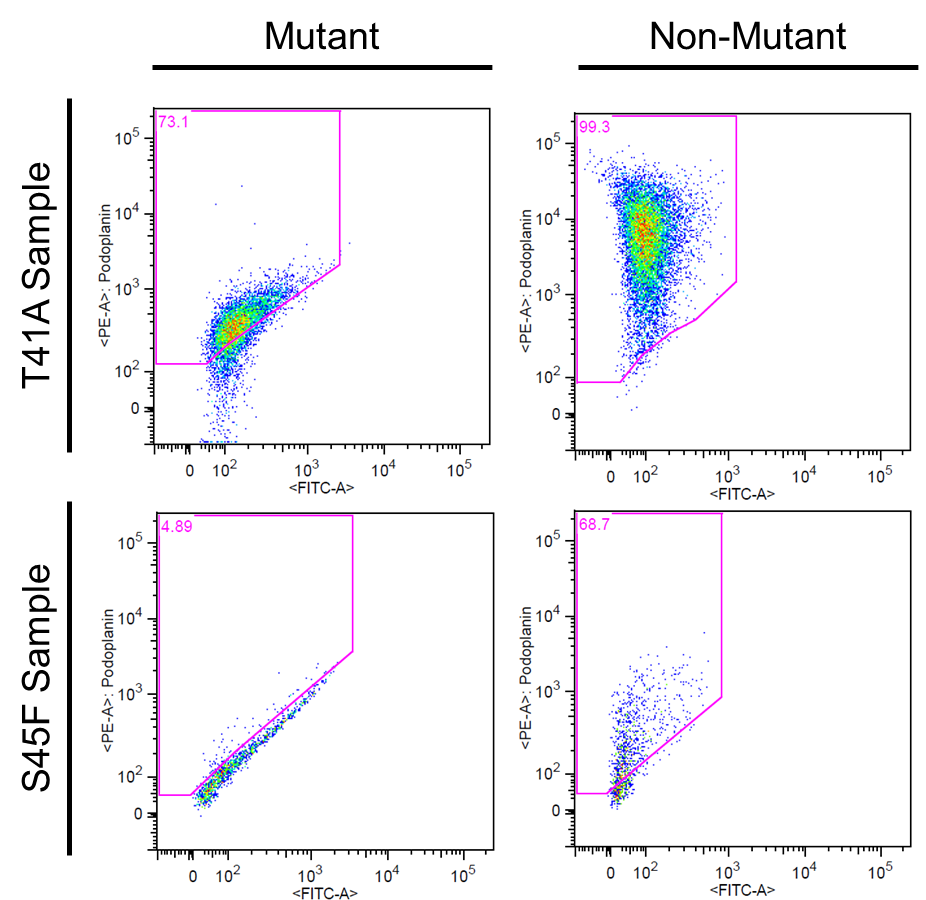


Supplementary Figure S4. Podoplanin-based flow cytometry of mutant and non-mutant colonies.

Supplement: Supplementary Figure S4 — Podoplanin-based flow cytometry of mutant and non-mutant colonies. [file crc-22-0403-s04.docx]
