# Supplementary material for: CD142 Identifies Neoplastic Desmoid Tumor Cells, Uncovering Interactions Between Neoplastic and Stromal Cells That Drive Proliferation
Source: Cancer Res Commun. 2023 Apr 25;3(4):697–708. doi: 10.1158/2767-9764.CRC-22-0403 (PMC10128091; doi:10.1158/2767-9764.CRC-22-0403)
Supplement: Supplementary Figure S5 — Measuring gene expression of F3 and PDPN after Wnt3a treatment of skin fibroblasts [file crc-22-0403-s05.docx]

Supplementary Figure S5. Measuring gene expression of *F3* and *PDPN* after Wnt3a treatment of skin fibroblasts. n = 3 experimental replicates. * P < 0.05.
