# Supplementary material for: CD142 Identifies Neoplastic Desmoid Tumor Cells, Uncovering Interactions Between Neoplastic and Stromal Cells That Drive Proliferation
Source: Cancer Res Commun. 2023 Apr 25;3(4):697–708. doi: 10.1158/2767-9764.CRC-22-0403 (PMC10128091; doi:10.1158/2767-9764.CRC-22-0403)
Supplement: Supplementary Figure S6 — Correlation between estimated mutation frequency from Sanger sequencing and percent CD142-positive cells in heterogeneous desmoid tumor samples [file crc-22-0403-s06.docx]

~~
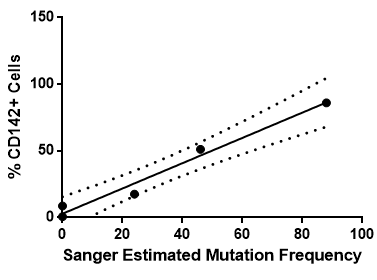
~~

Supplementary Figure S6. Correlation between estimated mutation frequency from Sanger sequencing and percent CD142-positive cells in heterogeneous desmoid tumor samples.

Pearson correlation coefficient r = 0.9874. P = 0.0017
