# Supplementary material for: CD142 Identifies Neoplastic Desmoid Tumor Cells, Uncovering Interactions Between Neoplastic and Stromal Cells That Drive Proliferation
Source: Cancer Res Commun. 2023 Apr 25;3(4):697–708. doi: 10.1158/2767-9764.CRC-22-0403 (PMC10128091; doi:10.1158/2767-9764.CRC-22-0403)
Supplement: Supplementary Figure S7 — Morphological differences in mutant and non-mutant cells isolated by FACS sorting [file crc-22-0403-s07.docx]

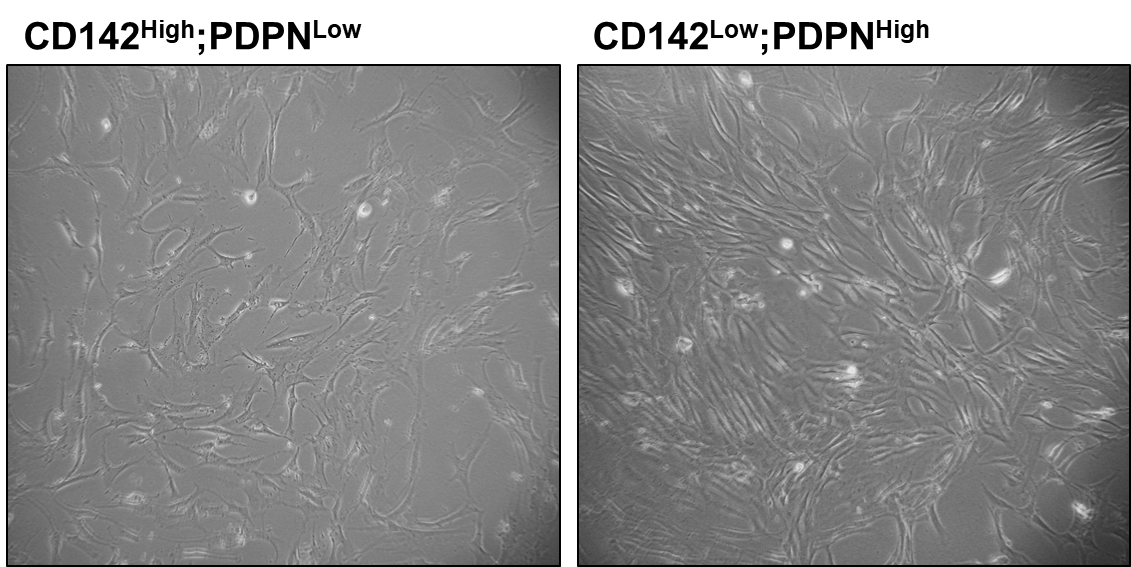


Supplementary Figure S7. Morphological differences in mutant and non-mutant cells isolated by FACS sorting.
