# Supplementary material for: CD142 Identifies Neoplastic Desmoid Tumor Cells, Uncovering Interactions Between Neoplastic and Stromal Cells That Drive Proliferation
Source: Cancer Res Commun. 2023 Apr 25;3(4):697–708. doi: 10.1158/2767-9764.CRC-22-0403 (PMC10128091; doi:10.1158/2767-9764.CRC-22-0403)
Supplement: Supplementary Figure S8 — Co-culturing of mutant desmoid tumor cells with non-mutant fibroblasts increases their proliferation rate [file crc-22-0403-s08.docx]

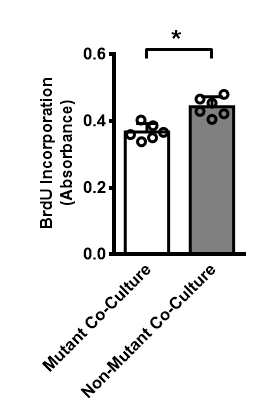


Supplementary Figure S8. Co-culturing of mutant desmoid tumor cells with non-mutant fibroblasts increases their proliferation rate. Data presented as mean + 95% confidence intervals. Circles indicate individual datapoints. n = 6 experimental replicates. * two-sample t-test P < 0.05.
