# Supplementary material for: CD142 Identifies Neoplastic Desmoid Tumor Cells, Uncovering Interactions Between Neoplastic and Stromal Cells That Drive Proliferation
Source: Cancer Res Commun. 2023 Apr 25;3(4):697–708. doi: 10.1158/2767-9764.CRC-22-0403 (PMC10128091; doi:10.1158/2767-9764.CRC-22-0403)
Supplement: Supplementary Figure S9 — Cytokine antibody array to identify differentially secreted factors from isolated mutant and non-mutant cells. [file crc-22-0403-s09.docx]

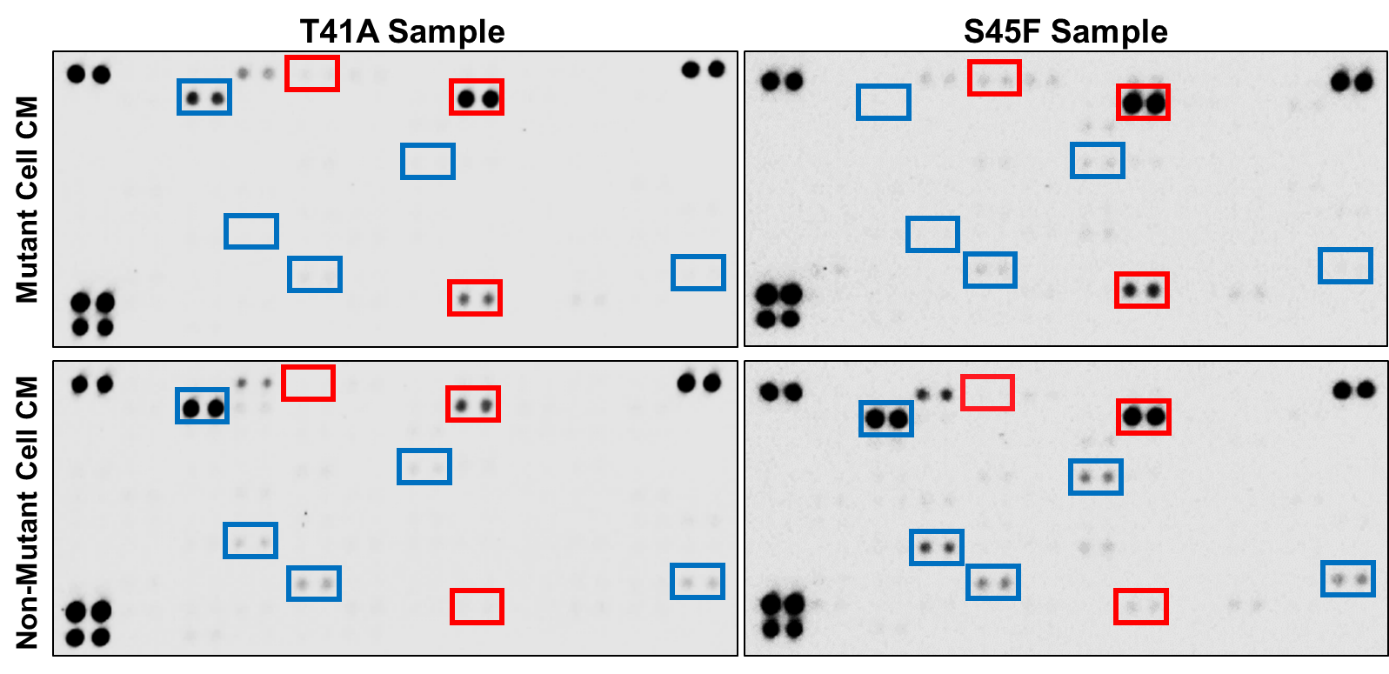


Supplementary Figure S9. Cytokine antibody array to identify differentially secreted factors from isolated mutant and non-mutant cells.

Duplicates in boxes indicates consistent trend. Red boxes indicate higher in mutant conditioned media, and blue boxes indicates higher in non-mutant conditioned media. See Supplementary Figure S10 for protein identity and densitometry.
