# Supplementary material for: CD142 Identifies Neoplastic Desmoid Tumor Cells, Uncovering Interactions Between Neoplastic and Stromal Cells That Drive Proliferation
Source: Cancer Res Commun. 2023 Apr 25;3(4):697–708. doi: 10.1158/2767-9764.CRC-22-0403 (PMC10128091; doi:10.1158/2767-9764.CRC-22-0403)
Supplement: Supplementary Figure S11 — Histologic appearance of two cases of desmoid tumors with different patterns of CD142 expression [file crc-22-0403-s11.docx]

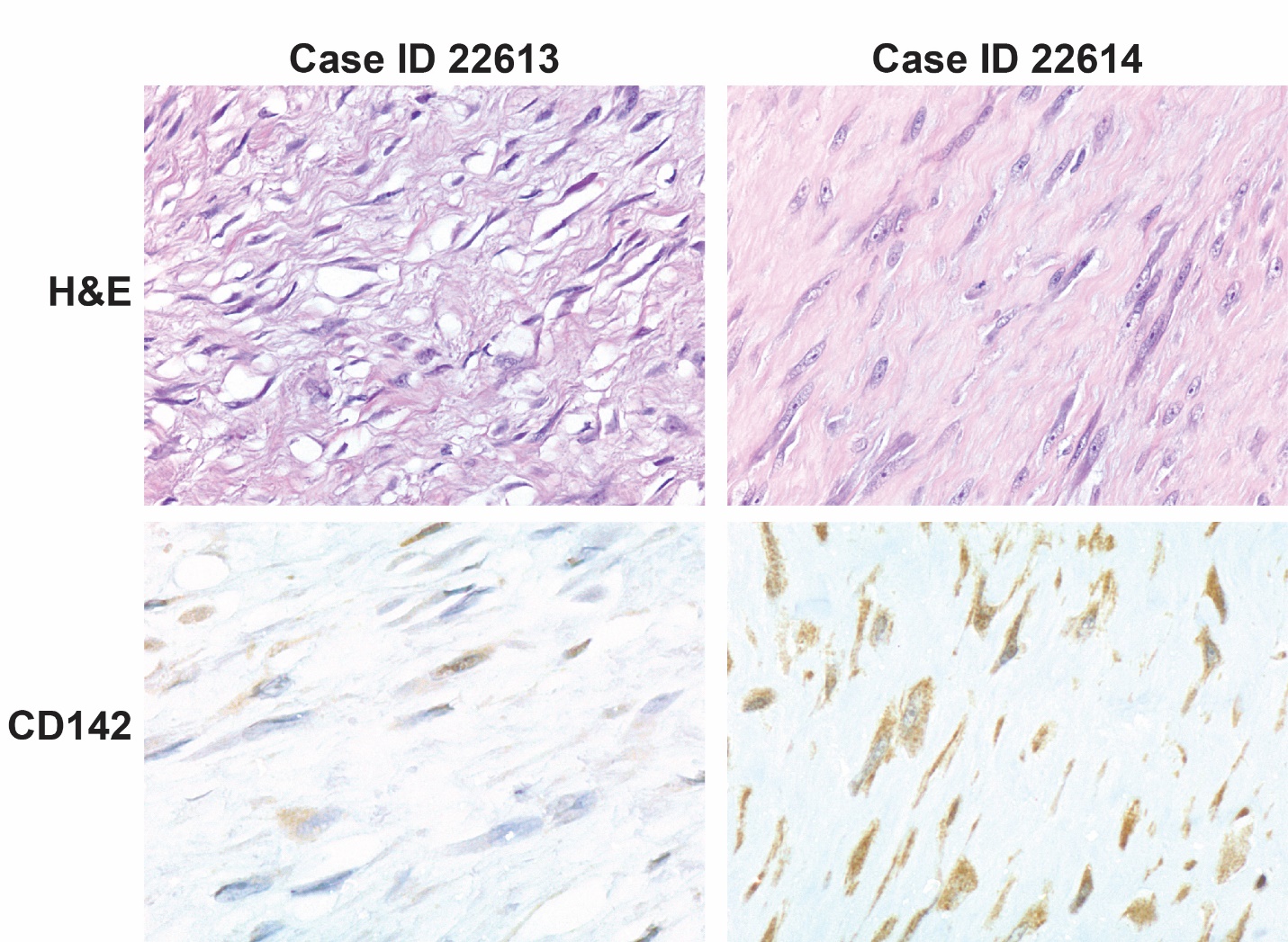


**Supplementary Figure S11. Histologic appearance of two cases of desmoid tumors with different patterns of CD142 expression.** Representative H&E staining in the first row shows bland spindle cell proliferation typical of desmoid tumors in both cases. Immunohistochemical staining in the second row shows a desmoid tumor with expression of CD142 in approximately 30% of cells (case ID 22613) and a desmoid tumor with expression of CD142 in all cells (case ID 22614). Total magnification for H&E - 300x and for CD142 staining - 600x. (H&E – hematoxylin and eosin).
