# Supplementary material for: CD142 Identifies Neoplastic Desmoid Tumor Cells, Uncovering Interactions Between Neoplastic and Stromal Cells That Drive Proliferation
Source: Cancer Res Commun. 2023 Apr 25;3(4):697–708. doi: 10.1158/2767-9764.CRC-22-0403 (PMC10128091; doi:10.1158/2767-9764.CRC-22-0403)
Supplement: Supplementary Figure S12 — Representative gating strategy [file crc-22-0403-s12.docx]

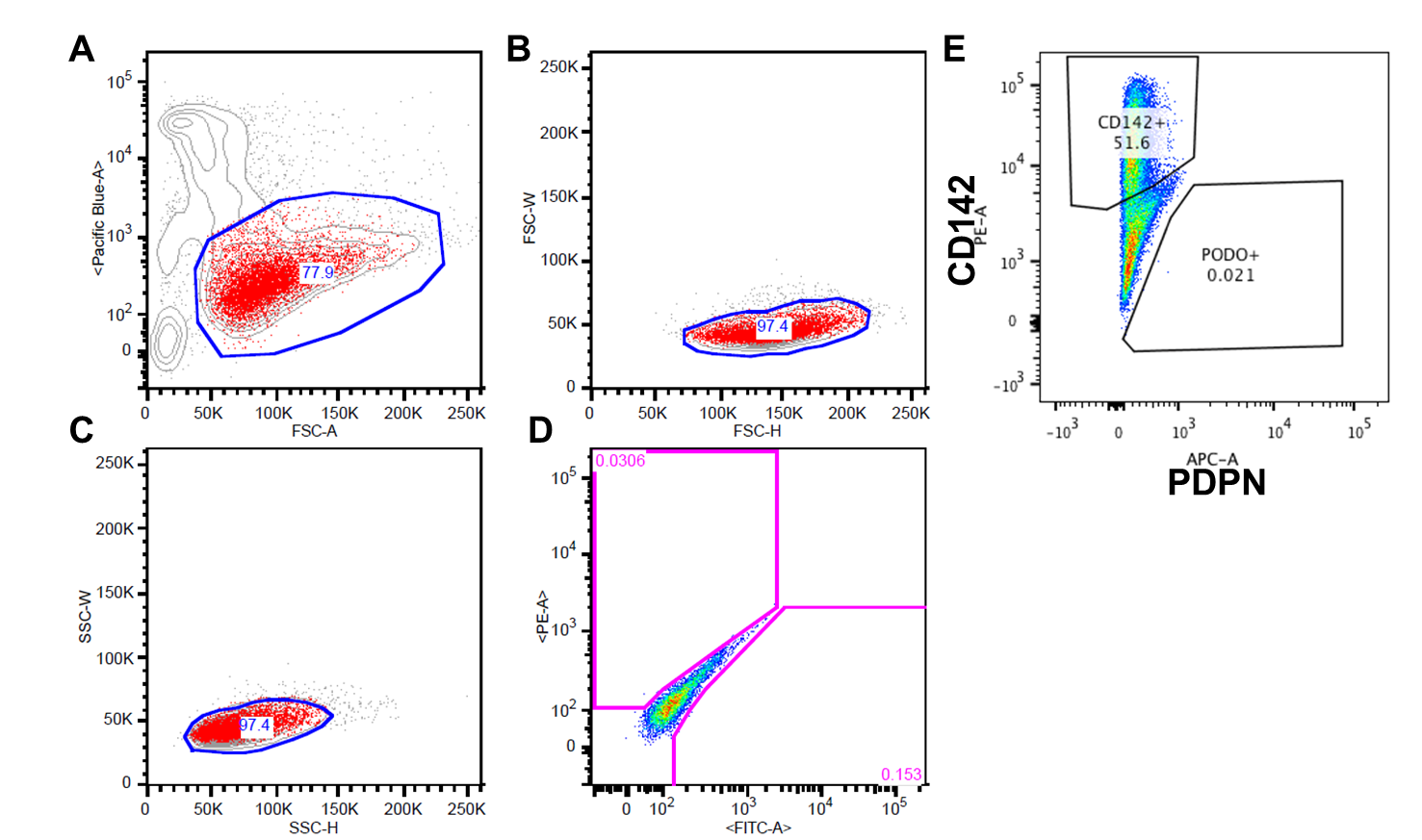


**Supplementary Figure S12. Representative gating strategy.** (A) Viable DAPI-negative cells were gated on forward scatter (FSC) and Pacific Blue channel, (B-C) Doublets were excluded using FSC and SSC height‐vs.‐width plots. (D) Positive gate was determined based on unstained control (here FITC is an empty channel). (E) Example of fluorescence-minus-one control for CD142-PDPN double-staining experiment.
