# Supplementary material for: CD142 Identifies Neoplastic Desmoid Tumor Cells, Uncovering Interactions Between Neoplastic and Stromal Cells That Drive Proliferation
Source: Cancer Res Commun. 2023 Apr 25;3(4):697–708. doi: 10.1158/2767-9764.CRC-22-0403 (PMC10128091; doi:10.1158/2767-9764.CRC-22-0403)
Supplement: Supplementary Table S2 — Enriched markers in mutant and non-mutant colonies from our high throughput surface antigen screen. [file crc-22-0403-s14.docx]

Supplementary Table S2. Enriched markers in mutant and non-mutant colonies from our high throughput surface antigen screen.

| *Mutant-enriched markers in T41A experiment* | | | | |
| --- | --- | --- | --- | --- |
| **Antibody** | **MUT_S45F POSITIVE% (MFI)** | **WT_S45F POSITIVE% (MFI)** | **MUT_T41A POSITIVE% (MFI)** | **WT_T41A POSITIVE% (MFI)** |
| CD90 | 97.6 (1176) | 91.7 (11693) | 81.8 (9153) | 99.3 (865) |
| **CD142*** | 92.4 (769) | 0.0 (0.1) | 92.6 (670) | 1.9 (278) |
| CD273 | 8.1 (133) | 3.2 (100) | 72.0 (358) | 6.5 (239) |
| SSEA-4 | 0.9 (116) | 1.6 (101) | 65.9 (459) | 1.3 (235) |
|  |  |  |  |  |
| *Mutant-enriched markers in S45F experiment* | | | | |
| **Antibody** | **MUT_S45F POSITIVE% (MFI)** | **WT_S45F POSITIVE% (MFI)** | **MUT_T41A POSITIVE% (MFI)** | **WT_T41A POSITIVE% (MFI)** |
| CD49b | 99.8 (2880) | 94.0 (284) | 99.6 (4418) | 99.2 (1270) |
| **CD142*** | 92.4 (769) | 0.0 (0.1) | 92.6 (670) | 1.9 (278) |
| CD252 | 79.8 (501) | 0.8 (92.8) | 61.0 (514) | 26.1 (260) |
| CD351 | 54.6 (206) | 9.6 (110) | 79.1 (450) | 86.3 (342) |
|  |  |  |  |  |
| *Non-mutant-enriched markers in T41A experiment* | | | | |
| **Antibody** | **MUT_S45F POSITIVE% (MFI)** | **WT_S45F POSITIVE% (MFI)** | **MUT_T41A POSITIVE% (MFI)** | **WT_T41A POSITIVE% (MFI)** |
| CD10 | 99.2 (964) | 90.7 (425) | 90.5 (737) | 99.8 (8422) |
| CD54 | 22.8 (356) | 38.1 (173) | 6.4 (389) | 90.7 (835) |
| **Podoplanin*** | 4.9 (244) | 68.7 (255) | 73.1 (410) | 99.3 (5864) |
| CD222 | 0.6 (165) | 1.6 (109) | 9.2 (247) | 52.1 (230) |
| CD61 | 1.1 (126) | 0.8 (80.7) | 0.4 (237) | 66.8 (335) |
| CD201 | 0.0 (0.1) | 0.2 (2101) | 0.8 (374) | 70.6 (390) |
|  |  |  |  |  |
| *Non-mutant-enriched markers in S45F experiment* | | | | |
| **Antibody** | **MUT_S45F POSITIVE% (MFI)** | **WT_S45F POSITIVE% (MFI)** | **MUT_T41A POSITIVE% (MFI)** | **WT_T41A POSITIVE% (MFI)** |
| **Podoplanin*** | 4.9 (244) | 68.7 (255) | 73.1 (410) | 99.3 (5864) |
|  |  |  |  |  |

MFI: Median Fluorescence Intensity
* Indicates a common marker in both experiments and was selected for further study.
