# Supplementary material for: CD142 Identifies Neoplastic Desmoid Tumor Cells, Uncovering Interactions Between Neoplastic and Stromal Cells That Drive Proliferation
Source: Cancer Res Commun. 2023 Apr 25;3(4):697–708. doi: 10.1158/2767-9764.CRC-22-0403 (PMC10128091; doi:10.1158/2767-9764.CRC-22-0403)
Supplement: Supplementary Table S3 — CD142 immunohistochemical analysis in scar tissue cores. Results from duplicate cores per do are shown when available. [file crc-22-0403-s15.docx]

**Supplementary Table S3. CD142 immunohistochemical analysis in scar tissue cores.** Results from duplicate cores per do are shown when available.

| **Donor ID** | **% of Positive Cells** | **Stain Intensity** |
| --- | --- | --- |
| 22595 | 95 | 3+ |
| 22596 | 95 | 3+ |
| 22598 | 95 | 3+ |
| 22599 | 95 | 3+ |
|  | 95 | 3+ |
| 22600 | 50 | 3+ |
|  | 95 | 3+ |
| 22601 | 95 | 3+ |
| 22602 | 95 | 3+ |
| 22603 | 90 | 3+ |
|  | 70 | 3+ |
| 22604 | 60 | 3+ |
|  | 60 | 3+ |
| 22605 | 0 | 0 |
