# Supplementary material for: CD142 Identifies Neoplastic Desmoid Tumor Cells, Uncovering Interactions Between Neoplastic and Stromal Cells That Drive Proliferation
Source: Cancer Res Commun. 2023 Apr 25;3(4):697–708. doi: 10.1158/2767-9764.CRC-22-0403 (PMC10128091; doi:10.1158/2767-9764.CRC-22-0403)
Supplement: Supplementary Table S4 — Summary of double-color cell sorting experiments. [file crc-22-0403-s16.docx]

**Supplementary Table S4. Summary of double-color cell sorting experiments.**

| **Test ID** | **Primary Estimated Mutation %** | **CD142^High^;PDPN^Low^**  **(Mutation)** | **CD142^Low^;PDPN^High^**  **(Mutation)** |
| --- | --- | --- | --- |
| #1 | 88% (S45F) | 84.6% (S45F) | 0% (N/D) |
| #2 | 46% (S45F) | 47.5%  (S45F) | 21.9%  (WT) |
| #3 | 24% (T41A) | 14.8%  (T41A) | 64.2%  (WT) |
| #4 | WT | 0%  (N/D) | 98%  (WT) |

N/D = not determined.
