# Supplementary material for: CD142 Identifies Neoplastic Desmoid Tumor Cells, Uncovering Interactions Between Neoplastic and Stromal Cells That Drive Proliferation
Source: Cancer Res Commun. 2023 Apr 25;3(4):697–708. doi: 10.1158/2767-9764.CRC-22-0403 (PMC10128091; doi:10.1158/2767-9764.CRC-22-0403)
Supplement: Supplementary Table S5 — Primers used in real-time quantitative PCR experiments. [file crc-22-0403-s17.docx]

**Supplementary Table S5. Primers used in real-time quantitative PCR experiments.**

| **Target Gene** | **Forward** | **Reverse** |
| --- | --- | --- |
| *AXIN2* | AAGCAGCGGTGCTGTGTGGC | AGGGTGTGGCTCCCGTCTGA |
| *CD142/F3* | TGGCACGGGTCTTCTCCTAC | GGCTGTCCGAGGTTTGTCTC |
| *PDPN* | CGGGAAGGTACTCGCCCTAA | GGTCATCTTCTCCCACGAGC |
| *THBS1* | GCACACAGGAAACACCCCT | CACCACTCTAATGAAACCCGTC |
| *CHI3L1* | CAGATGCCCTTGACCGCTT | AGGTTGGGGTTCCTGTTCTTG |
| *DKK1* | CACGCTATGTGCTGCCCC | ACCTTCTTGTCCTTTGGTGTGA |
| *ANGPT1* | GGGAACCGAGCCTATTCACA | GCATCAAACCACCATCCTCC |
| *IGFBP3* | GCCGTAGAGAAATGGAAGACAC | GGAAGGGCGACACTGCTTTT |
| *PTX3* | GCGGCTACCACTGTTGAGATG | CTCCCAGAGAAGGCTAATGTTTC |
| *CXCL12* | TGCCTCAGCGACGGGAA | TGTCTGTTGTTGTTCTTCAGCC |
| *CCL2* | AAGCAGAAGTGGGTTCAGGATT | CTTGGGTTGTGGAGTGAGTGT |
| *HPRT* | CCCTGGCGTCGTGATTAGTG | GCCTCCCATCTCCTTCATCACA |
